# Nanozymes for Advanced Hemoglobin‐Based Oxygen Carriers: Applications in Blood Substitution, Wound Healing, Antitumor Therapy, and Beyond

**DOI:** 10.1002/advs.202524094

**Published:** 2026-05-20

**Authors:** Despoina Douka, Ana María Pablo‐Sainz‐Ezquerra, Eva Jakljevič, Niki Ktena, Leticia Hosta‐Rigau

**Affiliations:** ^1^ Department of Health Technology Technical University of Denmark Kongens Lyngby Denmark

**Keywords:** blood substitutes, cancer, hemoglobin‐based oxygen carriers, nanozymes, wound healing

## Abstract

Oxygen delivery and reactive oxygen species regulation are tightly linked in physiology, with red blood cells serving both as oxygen transporters and as a key antioxidant barrier. This dual function has motivated decades of research into hemoglobin (Hb)‐based oxygen carriers (HBOCs), yet conventional designs remain susceptible to oxidative damage and lack the redox machinery of erythrocytes. In parallel, nanozymes—nanomaterials with enzyme‐mimetic catalytic activities—have emerged as powerful tools in redox medicine. Their integration with HBOCs has created a new class of self‐protecting, multifunctional, and stimuli‐responsive oxygen therapeutics. This review summarizes the foundations and biomedical potential of nanozyme‐integrated HBOCs and Hb‐derived nanozymes. We outline how metal, carbon, and metal–organic framework nanozymes replicate key antioxidant functions, including superoxide dismutase‐, catalase‐, and peroxidase‐like activity. Embedding such nanozymes into HBOCs imparts intrinsic oxidative protection and additional capabilities such as carbonic anhydrase‐like catalysis, transforming passive oxygen carriers into intelligent blood substitutes. We then highlight applications beyond transfusion, including wound healing, where photothermal nanozyme–HBOC hybrids enable light‐controlled oxygen release and antibacterial action, and cancer therapy, where catalytic HBOCs alleviate tumor hypoxia and enhance radiotherapy, photothermal therapy, and photodynamic therapy. Finally, we discuss Hb‐derived and erythrocyte‐templated nanozymes for biosensing, radioprotection, and infection control.

## Graphical abstract

This review explores how hemoglobin‐based oxygen carriers (HBOCs) combined with nanozymes create multifunctional materials that deliver oxygen while maintaining redox homeostasis. Beyond artificial blood substitutes, these constructs enable wound healing with light‐triggered oxygen release, cancer therapy through enhanced oxygenation and reactive oxygen species modulation, and infection control. These nanozyme‐integrated HBOCs open new directions in oxygen‐regulating medicine and innovative therapeutic strategies.

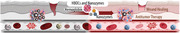

## 1 Introduction

Oxygen is vital to aerobic life as the terminal electron acceptor in cellular respiration, yet its partial reduction produces reactive oxygen species (ROS) such as superoxide radical anion (O_2_^•^^−^) and hydrogen peroxide (H_2_O_2_), which can damage lipids, proteins, and DNA [1]. Within the human body, red blood cells (RBCs) maintain a delicate equilibrium between oxygen delivery and redox control through hemoglobin (Hb) and an intracellular network of antioxidant enzymes. This fine balance has inspired decades of research into Hb‐based oxygen carriers (HBOCs), which are synthetic systems designed to transport oxygen in situations where donor blood is unavailable or contraindicated [2, 3, 4]. Beyond transfusion medicine, however, the unique oxygen‐binding properties of Hb, together with recent advances in nanozyme chemistry, have expanded the potential of HBOCs into broader biomedical territories, including wound healing, cancer therapy, and even the de novo synthesis of catalytic materials from Hb itself.

Nanozymes, which are inorganic or hybrid nanomaterials that mimic the catalytic activities of natural enzymes can display superoxide dismutase (SOD)‐, catalase (CAT)‐, or peroxidase‐like behavior, decomposing O_2_^•−^ and H_2_O_2_ with efficiencies comparable to their biological counterparts while retaining exceptional stability [5, 6, 7]. Unlike native enzymes, nanozymes can operate under harsh conditions, resist denaturation while also acting catalytically rather than stoichiometrically, providing sustained protection against oxidative stress [7]. The intersection of these two technologies, Hb‐based oxygen delivery and nanozyme‐based ROS regulation, offers a powerful, biomimetic solution to one of medicine's oldest challenges: how to deliver oxygen safely in the presence of oxygen‐derived radicals.

Integrating nanozymes into HBOCs transforms the design philosophy from structural mimicry to functional biomimicry. Instead of merely stabilizing Hb, these hybrid constructs actively mimic RBC physiology by coupling oxygen binding with catalytic detoxification of ROS. Pt, Ce oxide (CeO_2_), and Au‐based nanozymes have already demonstrated dual functionality: maintaining Hb in its ferrous (Fe^2+^) state while preventing oxidative degradation [8, 9, 10, 11].

Beyond antioxidation, many nanozymes possess photothermal or redox‐switchable properties that enable external control over oxygen release or catalytic activity, introducing a degree of spatiotemporal precision unattainable in earlier HBOC designs. The biomedical implications are profound. In blood substitution, nanozyme‐integrated HBOCs can serve as stable oxygen reservoirs with extended shelf lives and built‐in antioxidant protection. In wound healing, they can act as localized “oxygen depots” that restore normoxia to ischemic tissue while modulating inflammation and infection through controlled ROS generation or removal [12]. In cancer therapy, they can reoxygenate hypoxic tumors to sensitize them to radiotherapy (RT) or photodynamic therapy (PDT) while simultaneously catalyzing cytotoxic ROS production for synergistic tumor eradication [13, 14]. In each case, nanozymes confer catalytic resilience and multifunctionality, enabling HBOCs to operate as intelligent oxygen therapeutics rather than passive carriers.

A complementary frontier has also emerged: Hb‐related nanozymes, where Hb or RBC components serve not as oxygen carriers but as precursors or templates for catalytic nanomaterials. During thermal or chemical transformation, the Fe‐heme centers and amino acid residues of Hb act as intrinsic dopants, yielding Fe‐based carbonaceous nanozymes with remarkable peroxidase‐like activity and biocompatibility [15, 16, 17, 18]. These Hb‐derived catalysts close the conceptual loop by turning the molecule responsible for oxygen transport into the very material that catalyzes redox reactions, thus bridging sustainable nanomanufacturing with biomedical function.

Taken together, these advances highlight a rapidly evolving discipline at the interface of nanotechnology, catalysis, and bioinspired materials design. By merging the oxygen‐handling sophistication of Hb with the catalytic robustness of nanozymes, researchers are now constructing systems that can deliver, sense, and regulate oxygen and ROS within the same platform. Such multifunctional materials hold promise not only for emergency transfusion or ischemic rescue but also for chronic disease management, tissue regeneration, and precision oncology.

**SCHEME 1:**
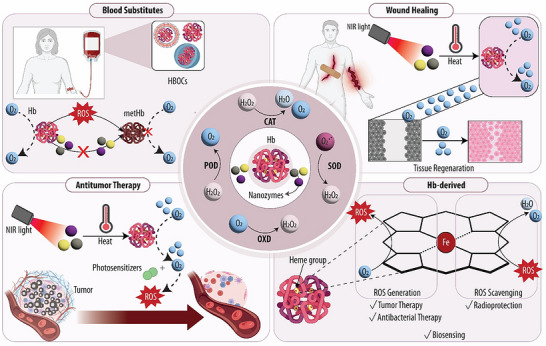
Multifunctional applications of hemoglobin (Hb)‐integrated nanozymes in biomedical therapy and regenerative medicine. Center: catalytic pathways of Hb‐nanozyme hybrids, highlighting superoxide dismutase (SOD), catalase (CAT), peroxidase (POD), and oxidase (OXD) activities that regulate the conversion between oxygen and reactive oxygen species (ROS) to maintain redox‐balanced microenvironments. Top left: incorporation of nanozymes into Hb‐based oxygen carriers (HBOCs) to protect Hb from oxidation into methemoglobin (metHb) and minimize ROS, thereby preserving oxygen‐carrying capacity. Top right: wound‐healing applications under near‐infrared (NIR) irradiation, where nanozyme‐mediated photothermal heating triggers Hb‐bound oxygen release and enhances tissue regeneration. Bottom left: antitumor strategies involving radiotherapy, photothermal or photodynamic therapy, in which nanozymes modulate intratumoral ROS levels. Simultaneously, oxygen released from Hb alleviates tumor hypoxia and promotes ROS generation by photosensitizers or thermal processes, thereby enhancing oxidative stress and improving tumor cell killing efficacy. Bottom right: Hb‐derived catalytic platforms enabling ROS generation (for tumor/antibacterial therapy), ROS scavenging (for radioprotection), and biosensing, driven by the Fe‐centered redox chemistry of the heme structure. Made and modified from BioRender.

This review surveys the emerging landscape of nanozyme‐enhanced HBOCs and Hb‐derived nanozymes, highlighting how catalytic nanochemistry is redefining oxygen therapeutics. We first discuss nanozyme integration strategies that endow HBOCs with antioxidant self‐defense for use as advanced blood substitutes. We then examine their translation into wound‐healing scaffolds and antitumor nanoplatforms and finally explore how Hb itself can be repurposed as a structural and chemical template for redox‐active nanozymes (Scheme 1). Together, these perspectives reveal how catalytic materials science is transforming the century‐old pursuit of artificial blood into a broader paradigm of adaptive, multifunctional, and self‐regulating oxygen medicine.

## 2 Nanozymes: Historical Roots, Mechanistic Insights, and Design Strategies

### 2.1 Definition and Historical Context

The conceptual roots of nanozymes lie in the 19th‐century foundations of catalysis and biocatalysis: catalysis was defined in 1835 as acceleration of reactions by substances that remain unchanged [19], and “enzyme” was coined in 1877 from the Greek *ενζυμον* (“in leaven”) during early fermentation studies [20]. Through the mid‐20th century, biocatalysis was dominated by natural enzymes, with parallel efforts to create artificial enzymes involving synthetic mimics (cyclodextrins, crown ethers, peptides, micelles, molecularly imprinted polymers) [21, 22], semi‐synthetic enzymes obtained by chemically modifying proteins [23], and nucleic‐acid‐based DNAzymes and abzymes [24, 25]. Despite progress, most artificial enzymes underperform natural counterparts in activity [7].

The nanozyme concept emerged when nanoscale matter itself was recognized as catalytically active. In 2004, Pasquato and Scrimin used the term “nanozyme” to name triazacyclononane‐functionalized Au nanoparticles (NPs) that catalyzed transphosphorylation [26]. Related uses described a Ga^3^^+^ supramolecular catalyst for acetal/ketal hydrolysis [27] and a CAT‐encapsulating cationic block copolymer [28], which are systems now viewed as nano‐immobilized catalysts rather than intrinsically active nanozymes [7]. The watershed came in 2007 with the discovery that iron oxide (Fe_3_O_4_) NPs intrinsically oxidize peroxidase substrates, such as 3,3′,5,5,′‐tetramethylbenzidine (TMB), 3,3′‐diaminobenzidine and 1,2‐diaminobenzene (OPD) in the presence of H_2_O_2_, revealing horseradish peroxidase (HRP)‐like activity [29]. The field then expanded rapidly, prompting the term “nanozymology” with more than 200 research groups around the world working on nanozyme research [30].

Reflecting this evolution, a 2021 definition formalized nanozymes as nanomaterials that convert enzyme substrates to products with enzymatic kinetics (e.g., Michaelis–Menten) under physiologically relevant conditions, even if their molecular mechanisms differ from native enzymes [31]. To improve nomenclature fidelity, “enzyme‐like” describes nanozymes sharing the same substrates/products as enzymes, whereas “enzyme‐mimicking” refers to efforts reproducing structural/functional motifs of enzymatic active sites without necessarily replicating the full catalytic mechanism [6]. Importantly, nanozymes add nanostructure‐confined active sites, tunable size/morphology, and unique nano‐physicochemical properties (e.g., superparamagnetism, photothermal effects), expanding biocatalysis across biomedicine, environmental management, and agriculture [7].

### 2.2 Mechanistic Principles, Design, and Engineering of Nanozymes

Nanozymes are nanoscale catalytic materials whose enzymatic activity arises from surface‐confined and structure‐dependent effects. Their high surface‐to‐volume ratios, heterogeneous active sites, and nanoconfined electronic structures facilitate substrate adsorption and activation, enabling reactions analogous to those of natural enzymes [6, 7]. Importantly, their turnover is an intrinsic property of the nanostructure itself and does not originate from leached metal ions or appended molecular catalysts [7, 32]. However, not all nanozyme‐mediated ROS scavenging reflects genuine catalytic cycling. While certain systems (e.g., Pt‐, Au‐, and CeO_2_‐based nanozymes) operate through reversible surface redox transitions that support sustained turnover, others undergo partial or complete redox consumption depending on pH and substrate flux. For example, at neutral pH, manganese dioxide (MnO_2_) can exhibit CAT‐like activity by decomposing H_2_O_2_ into molecular oxygen; however, in mildly acidic environment, MnO_2_ instead reacts with H_2_O_2_ and H^+^ to yield molecular oxygen and soluble Mn^2+^, resulting in the loss of true catalytic cycling and long‐term activity [33]. Distinguishing between catalytic ROS regulation and sacrificial redox buffering is therefore essential when evaluating nanozymes intended for biomedical use. In addition, the biological consequences of nanozyme activity depend critically on the specific reaction pathway involved, namely whether the material primarily eliminates ROS (e.g., O_2_^•^^−^ dismutation or H_2_O_2_ decomposition) or instead promotes ROS generation through peroxidase‐like processes. To clarify these mechanistic distinctions, Table 1 summarizes the major enzyme‐like catalytic activities exhibited by nanozymes, classifying them according to primary substrates, representative reactions, products, and biological roles. The table further highlights the functional contrast between antioxidant pathways (SOD‐ and CAT‐like) and ROS‐promoting pathways (peroxidase (POD)‐like), while noting that catalytic behavior is often context‐dependent and may shift with composition, oxidation state, and environmental parameters such as pH.

**TABLE 1:** Comparison of major enzyme‐like catalytic mechanisms exhibited by nanozymes. Activities are classified according to their primary substrates, representative reactions, products, and biological roles, highlighting the distinction between antioxidant, such as superoxide dismutase (SOD)‐ and catalase (CAT)‐like and reactive oxygen species (ROS)‐promoting (peroxidase (POD)‐like) pathways. Representative nanozyme systems are listed to illustrate material‐specific examples, noting that catalytic behavior may depend on composition, oxidation state, and environmental conditions (e.g., pH).

Enzyme‐like activity	Substrate	Reaction	Products	Biological role	Representative Nanozymes
SOD‐like	O_2_^•−^	2O_2_^•−^+ 2H^+^→ H_2_O_2_ + O_2_	H_2_O_2_ + O_2_	Antioxidant	PtNPs, CeO_2_NPs, AuNPs
CAT‐like	H_2_O_2_	2H_2_O_2_ → 2H_2_O + O_2_	H_2_O + O_2_	Antioxidant	PtNPs, CeO_2_NPs, AuNPs, MnO_2_NPs (neutral pH)
POD‐like	H_2_O_2_	H_2_O_2_ + e^−^ donor→ •OH	•OH radicals	ROS generation	Fe_3_O_4_NPs, MnO_2_NPs(acidic pH)

Catalytic reactions predominantly occur at specific surface interfaces, such as crystal planes, unsaturated coordination sites, heterostructure boundaries, or defect regions (steps, edges, and corners), where local geometry and electronic density govern the activation of reactants [34]. Since multiple active site types coexist within a single NP, a given nanozyme can mediate multiple concurrent reactions. For example, it may decompose H_2_O_2_ into hydroxyl radicals (^•^OH) in a peroxidase‐like manner or convert it into molecular oxygen via a CAT‐like mechanism. This structural versatility underlies their broad functionality but also contributes to reduced reaction specificity. In contrast to natural enzymes, which are typically characterized by high substrate fidelity, nanozymes are substrate‐promiscuous, a property that favors cascade catalysis and synergistic reactions, yet complicates precise single‐pathway control [7, 30].

To harness and refine these catalytic behaviors, diverse nanomaterial frameworks have been engineered to exhibit enzyme‐like activity. Thousands of inorganic and organic nanostructures, from bulk NPs to single‐atom catalysts (SAzymes), have demonstrated such properties [5]. Four principal material classes dominate: metal‐, carbon‐, metal–organic framework (MOF)‐based, and SAzyme systems.

Among them, metal‐based nanozymes remain the most extensively investigated (∼2877 publications). These include Fe‐based families (Fe oxides, chalcogenides, phosphates, and Prussian blue analogues)) [35, 36] and non‐Fe systems such as noble metals (Au, Ag, Pt, Ir) and transition‐metal oxides/sulfides (Cu, V, Mn, Ce, Cd) [5, 37, 38, 39, 40]. Multimetallic architectures often outperform monometallic counterparts through synergistic and electronic coupling effects, highlighting the importance of deliberate control over composition, structure, and oxidation state [41, 42]. Carbon‐based nanozymes, first reported in 2010, encompass carbon nanotubes [43], graphene oxide [44], carbon nitride [45], carbon nanodots [46], fullerenes [47], and other related carbons [48, 49]. Their catalytic performance can be dramatically tuned by heteroatom doping (N, Fe, Co, Se, Ni), which modulates electronic density and creates enzyme‐like active sites, producing high‐efficiency multienzyme catalysts [50, 51, 52, 53]. MOF‐based nanozymes provide an additional level of tunability. Their highly porous, size‐uniform architecture enhances substrate diffusion and enables size‐selective catalysis [5]. The modular nature of MOFs, featuring adjustable metal nodes and organic linkers, allows fine‐tuning of biocompatibility, biodegradability, and functionality, making them particularly attractive for biosensing, biocatalysis, and therapeutic applications [54, 55]. At the atomic scale, SAzymes, which were first reported in 2019 [56], achieve nearly 100% atomic utilization and possess well‐defined catalytic centers. Most adopt metal‐nitrogen‐carbon coordination motifs, where the metal identity and oxidation state, nitrogen coordination, and carbon matrix collectively dictate activity and stability. By tailoring coordination number and geometry, researchers can precisely modulate turnover frequency and substrate selectivity [57]. Dual‐atom SAzymes further enhance reactivity through cooperative catalytic effects [58, 59].

Beyond composition, morphological and electronic tuning remains central to nanozyme design. Even within a fixed chemical composition, variations in particle size, shape, or valence state can dramatically influence both catalytic type and overall activity [36]. Modern designs increasingly emphasize multi‐enzyme‐like and cascade behaviors, with over 20 types of enzyme mimics (bi‐, tri‐, tetra‐, and penta‐enzyme systems) now described, particularly within the oxidoreductase family [5]. Such architectures can establish self‐sustained catalytic loops, continuously generating intermediate species to boost efficiency in sensing and therapeutic contexts. Simultaneously, the integration of optical, electrical, magnetic, thermal, and acoustic properties with catalytic functions has enabled photo‐, electro‐, or magnetically enhanced catalysis, as well as multimodal imaging and other hybrid biomedical functionalities [5].

Despite these advances, most nanozymes still display lower turnover rates and broader substrate ranges compared to natural enzymes. Bridging this gap requires ongoing efforts in active‐site precision engineering, selectivity tuning, and multi‐functionality optimization to achieve enzyme‐like efficiency with nanoscale robustness [30, 60, 61].

## 3 Nanozyme‐Integrated HBOCs Toward Advanced Blood Substitutes

The transfusion of donor blood, though life‐saving and routine, presents persistent logistical and safety challenges. Donated blood must be correctly typed and cross‐matched, has a short storage lifetime, and may transmit infections [62, 63]. These limitations become critical in emergency settings, resource‐limited environments, or in patients who refuse blood transfusions for religious reasons. Consequently, there is a critical need for an artificial “oxygen bridge” capable of temporarily replace or augment blood function [64]. HBOCs are among the most studied blood substitute candidates, capitalizing on the excellent oxygen‐binding properties of Hb [4].

Research on HBOCs has produced a variety of formulation strategies to package Hb for use as an RBC substitute. Traditional HBOC designs include chemical cross‐linking or polymerization of Hb, surface PEGylation, encapsulation in liposomes (forming so‐called “Hb‐vesicles”), polymer‐based NPs, hydrogels, and MOFs [62, 63, 65, 66]. The goals of these strategies are to prevent dissociation of Hb into dimers (which causes renal toxicity), prolong circulation time, and avoid extravasation through blood vessel walls. Encapsulation approaches create a physical barrier around Hb that can reduce nitric oxide (NO)‐scavenging [67]. Being an important vasodilator, NO scavenging results in dangerous vasoconstriction and hypertension. Encapsulating Hb also shields the protein from rapid degradation while its confinement within liposomal or polymeric NPs increases its size and confers a negatively charged exterior (similar to albumin or cell membranes), which helps keep the HBOC within the vasculature.

**SCHEME 2:**
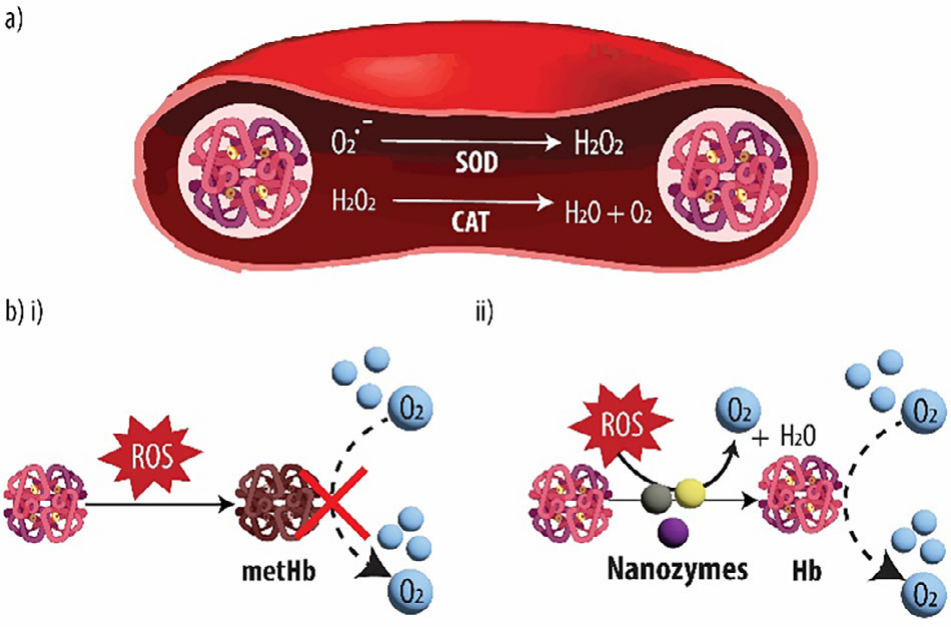
(a) Native red blood cells contain endogenous antioxidant enzymes that regulate reactive oxygen species (ROS) generated during hemoglobin (Hb) oxygen transport. Superoxide dismutase (SOD) converts superoxide radicals (O_2_^•−^) into hydrogen peroxide (H_2_O_2_), which is subsequently decomposed by catalase (CAT) into water (H_2_O) and molecular oxygen (O_2_), thereby maintaining redox balance and protecting Hb from oxidative damage. (b) Oxidative challenges in Hb‐based oxygen carriers (HBOCs). (i) In the absence of antioxidant protection, ROS accumulation promotes oxidation of Hb to methemoglobin (metHb), impairing oxygen‐binding capacity and oxygen delivery. (ii) Incorporation of nanozymes with SOD‐ and CAT‐like activities enables catalytic ROS scavenging, converting ROS into harmless products and regenerating O_2_, thereby preserving Hb in its functional state and sustaining oxygen transport.

While these engineering solutions tackle some HBOC issues, oxidative stress remains a major challenge. Hb outside the protective RBC environment is prone to autoxidation (Fe^2+^ to Fe^3+^), forming methemoglobin (metHb) that cannot carry oxygen (Scheme 2). The autoxidation process generates O_2_^•^^−^, which dismutates into H_2_O_2_. These ROS then further oxidize Hb and can damage tissues. In native RBCs, an elegant enzymatic antioxidant system (including SOD and CAT) rapidly neutralizes O_2_^•^^−^ and H_2_O_2_, protecting Hb and the surrounding cells. By contrast, most HBOCs lack an intrinsic antioxidant machinery and therefore suffer faster oxidative degeneration and associated side effects. Early attempts to address this issue tethered natural antioxidant enzymes directly to HBOCs. For example, Chang et al. conjugated polymerized Hb with the SOD and CAT enzymes, successfully reducing Hb's autoxidation rate [68]. Similarly, Kluger et al. constructed an Hb‐SOD fusion protein that inhibited metHb formation [69] and Silaghi‐Dumitrescu et al. incorporated a non‐heme Fe enzyme (rubrerythrin) into Hb resulting in a Hb‐enzyme co‐polymer with dual oxygen‐carrying and antioxidant activity [70]. These enzyme‐based HBOCs demonstrated the principle that concurrent oxygen transport and ROS scavenging is achievable. However, biological enzymes present issues: each enzyme targets a specific ROS, can be immunogenic, and tends to denature or lose activity over time in circulation [71]. This creates a need for more robust, broad‐spectrum solutions to protect HBOCs from oxidative damage. To contextualize the evolution of antioxidant strategies in HBOCs, Table 2 compares early enzyme‐conjugated systems with more recent nanozyme‐integrated designs. The upper section summarizes formulations incorporating natural antioxidant enzymes, while the lower section highlights nanozyme‐based platforms illustrating the progression toward more robust and multifunctional constructs.

**TABLE 2:** Comparison of enzyme‐conjugated and nanozyme‐integrated hemoglobin (Hb)‐based oxygen carriers (HBOCs). The upper section summarizes early systems incorporating natural antioxidant enzymes, including superoxide dismutase (SOD), catalase (CAT), and peroxidase (POD), into polymerized or crosslinked Hb formulations. The lower section highlights nanozyme‐based strategies employing Pt nanoparticles (PtNPs), cerium oxide NPs (CeO_2_‐NPs), Au‐NPs, Au nanoclusters (AuNCLs), nitroxide radicals and zeolitic imidazolate framework‐8 NPs (ZIF‐8 NPs). Reported enzyme‐like activities include superoxide dismutase (SOD)‐like, catalase (CAT)‐like, and carbonic anhydrase (CA)‐like functions.

Biological enzymes	HBOC	Integration strategy	Primary function	Reference
SOD and CAT	Polymerized Hb (PolyHb)	Conjugation of SOD and CAT with PolyHb	Reduction of Hb autoxidation rate	68
SOD	Intramolecularly crosslinked Hb	Conjugation of crosslinked Hb with crosslinked SOD	Inhibition of metHb formation	69
Non‐heme POD (Rubrerythrin)	Polymerized Hb	Hb copolymerized with rubrerythrin	Enhanced POD activity Lower levels of free radical	70

Nanozyme	HBOC	Integration strategy	Enzyme activity	Primary function	Reference
PtNPs	Hb‐(HSA)_3_	PtNPs embedded in HSA shell	SOD, CAT	ROS scavenging Antioxidant Hb protection	10
CeO_2_NPs	Hb^PDA^‐coated PLGA NPs (PLGA/Hb^PDA^)	CeO_2_NPs‐coated PLGA/Hb^PDA^	SOD, CAT	ROS scavenging	8
	Hb‐loaded PLGA NPs	CeO_2_NPs‐coated Hb‐loaded PLGA NPs	SOD, CAT	ROS scavenging	96
AuNPs	PCN‐333 NPs (Post encapsulated Hb)	AuNPs‐loaded PCN‐333 NPs (AuNPs@PCN‐333 NCs)	SOD, CAT	Recyclable ROS scavenging Antioxidant Hb protection	9
Au nanoclusters (AuNCLs)	Composite of Hb and Au nanoclusters (Hb@AuNCLs)		CAT	Recyclable ROS scavenging Antioxidant Hb protection	11
Nitroxides	PEGylated Hb	Polynitroxylated PEGylated Hb (PNPH)	SOD, CAT	ROS scavenging	110
ZIF‐8 NPs	Hb‐loaded ZIF‐8 NPs (Hb@ZIF‐8 NPs)		CA	CO_2_ decrease Buffering of pH	113

Abbreviations: PolyHb: polymerized Hb; HSA: human serum albumin; PLGA: poly(lactic‐co‐glycolic acid); PDA: polydopamine; Hb^PDA^: PDA‐coated Hb; PEG: polyethylene glycol; PNPH: polynitroxylated PEGylated Hb; PCN: porous coordination network; ROS: reactive oxygen species; CO_2_: carbon dioxide.

**FIGURE 1:**
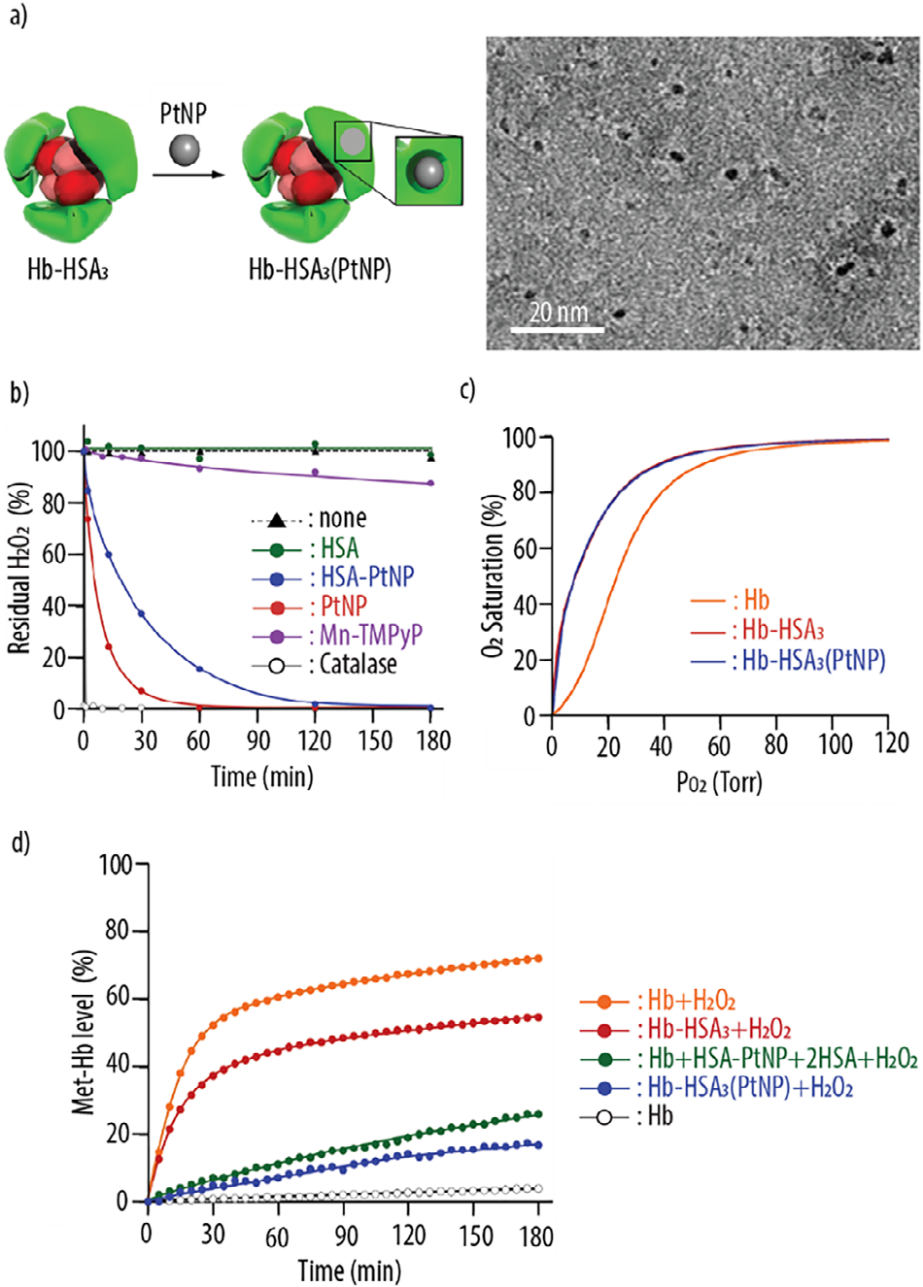
(a) Schematic illustration of hemoglobin (Hb)‐human serum albumin (HSA)‐Pt nanoclusters (Hb‐HSA_3_(PtNP)): Hb core is coated with three HSA molecules (Hb‐HSA_3_), which binds ultra‐small Pt nanoparticles (PtNP). Right: TEM image of HSA containing PtNP (HSA‐PtNP) negatively stained with 1% uranyl acetate. (b) Time‐dependent residual hydrogen peroxide (H_2_O_2_) (%) in 0.1 mm H_2_O_2_ at 25°C in the presence of no enzyme mimic or catalytic agent (none), HSA, HSA‐PtNP, PtNP, manganese(III) tetrakis(1‐methyl‐4‐pyridyl)porphyrin (Mn‐TMPyP), and catalase. (c) O_2_ equilibrium curves of Hb‐HSA_3_ and Hb‐HSA_3_(PtNP) in phosphate‐buffered saline (PBS, pH 7.4) at 37°C, showing O_2_ saturation (%) as a function of partial pressure of oxygen (P_O2_). (d) Time course of methemoglobin (metHb) generation for Hb‐HSA_3_, Hb‐HSA_3_(PtNP) and native Hb in presence of 20 µm H_2_O_2_ at 25°C ([Hb] = 10 µm). Adapted with permission [10]. Copyright 2014 PLoS One.

Nanozymes have emerged as an innovative solution to this problem. A landmark advancement was achieved by Komatsu and co‐workers, who first introduced nanozyme chemistry into HBOCs [10]. They developed a covalent Hb‐albumin cluster in which a central Hb molecule is shielded by three human serum albumin (HSA) molecules (Hb‐(HSA)_3_), a design that improves colloidal stability by leveraging albumin's large size and negative charge to limit Hb leakage from the vasculature [72, 73, 74]. PtNPs were subsequently incorporated into the albumin periphery of the cluster, yielding an HBOC with built‐in antioxidant enzyme mimics (Figure 1a) [10]. Ultrasmall citrate‐reduced PtNPs (∼1.8 nm) were embedded within the protein matrix, with structural analyses indicating accommodation within positively charged clefts of HSA via electrostatic interactions [75, 76]. The resulting HSA‐PtNP complexes displayed strong SOD‐ and CAT‐like activities, with O_2_^•^^−^ dismutation and H_2_O_2_ decomposition efficiencies comparable to or exceeding those of natural or synthetic enzyme mimics [77, 78, 79]. Integration of these nanozymes into the Hb–(HSA)_3_ cluster produced the Hb‐(HSA(PtNP))_3_ hybrid, in which the Hb core enables oxygen transport while the PtNP‐functionalized HSA shell catalytically removes O_2_^•^^−^ and H_2_O_2_ (Figure 1b,c). This architecture markedly enhanced the oxidative stability: after 180 min in 20 µm H_2_O_2_ solution, only 17% metHb was formed, compared to 72% for native Hb (Figure 1d). This work provided compelling proof‐of‐concept that direct nanozyme incorporation into HBOCs can mitigate one of the field's main hurdles, namely, the oxidative degradation of Hb. Such a configuration provides an artificial oxygen carrier with the potential to deliver oxygen to ischemic tissues while protecting Hb and surrounding cells from reperfusion‐induced oxidative damage.

Following the success of PtNPs‐HSA clusters, other nanozymes have been explored for integration into HBOCs. Our group developed a Hb‐based system incorporating CeO_2_ nanozymes [8]. CeO_2_NPs exploit the reversible Ce^3+^/Ce^4+^ redox couple to catalytically dismutate both H_2_O_2_ and O_2_^•^^−^, mimicking CAT and SOD activities [80]. The synthesized CeO_2_NPs were ultrasmall (2.2–2.6 nm), monodisperse, and negatively charged (zeta (ζ)‐potential = −26 ± 7 mV), with a crystalline fluorite structure. The resulting HBOC was a layer‐by‐layer (LbL) self‐assembled nanocarrier (NC) comprising a poly(lactide‐*co*‐glycolide) (PLGA) core sequentially coated with Hb and CeO_2_NPs and followed by a poly(ethylene glycol) (PEG) stealth layer. To preserve Hb structure and function during assembly, the protein was first coated with a polydopamine (PDA) layer, which promotes adhesion and biocompatibility [81, 82]. CeO_2_NPs exhibited strong and durable SOD‐ and CAT‐like activities, retaining catalytic performance for at least 21 days at 4°C—far exceeding the stability of native CAT. When integrated into the Hb‐loaded LbL NCs, CeO_2_NPs retained their catalytic performance over multiple ROS exposure cycles, confirming that the CeO_2_NPs acted catalytically rather than being consumed. PEGylation further enhanced stealth properties, reducing immunoglobulin G (IgG) adsorption from 91 to 11% and decreasing cellular uptake by up to 48%. The resulting CeO_2_‐based HBOCs thus combined long‐lasting antioxidant protection with low macrophage recognition, underscoring their promise as stable, ROS‐resistant oxygen carrier with potential extended circulation and storage lifetimes. However, at the time, direct assessment of whether nanozyme incorporation prolonged the functional Fe^2+^ state of Hb was hindered by PDA interference in UV–vis metHb quantification; this limitation is currently being addressed using Raman spectroscopy to monitor Hb oxidation states in complex assemblies.

To further enhance HBOC performance, biomimetic strategies have been combined with nanozyme‐based designs. Although PEGylation remains the gold standard for imparting stealth properties, the increasing prevalence of anti‐PEG antibodies has raised concerns regarding accelerated clearance by the mononuclear phagocyte system [83, 84, 85, 86]. Consequently, alternative surface modification approaches are actively being pursued, including coatings derived from RBC membranes [87, 88, 89, 90, 91]. RBCs achieve exceptional circulation lifetimes through a combination of surface proteins, glycans, and mechanical properties, with CD47 and terminal sialic acids playing key roles in immune evasion and lifespan regulation [92, 93, 94, 95]. Building on these principles, we coated Hb‐ and CeO_2_NPs‐loaded LbL NCs with erythrocyte membranes [96]. The resulting hybrids resisted protein adsorption, showed reduced uptake by endothelial cells, retained efficient oxygen transport, and preserved robust nanozyme activity across a wide temperature range. These systems also demonstrated excellent hemocompatibility and cellular viability, highlighting the advantages of combining nanozymes with biomimetic surface engineering.

**FIGURE 2:**
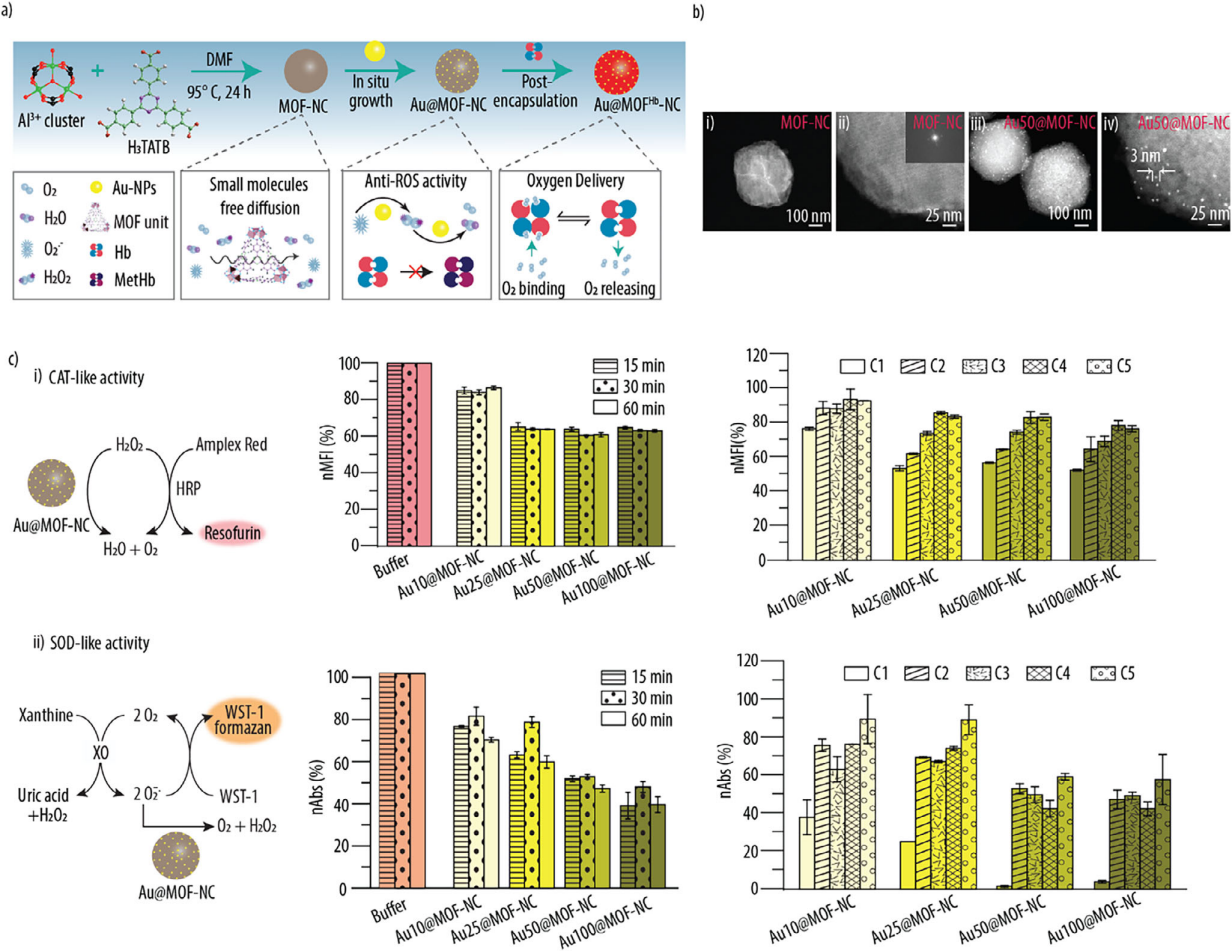
(a) Schematic illustration of the synthesis of hemoglobin (Hb)‐loaded metal‐organic framework (MOF) nanocarriers (NCs) (Hb@MOF‐NCs) incorporating Au‐based nanozymes (Au@MOF^Hb^‐NCs). MOF particles are formed from Al^3+^ clusters and the organic linker 4, 4′, 4″‐s‐triazine‐2, 4, 6‐triyl‐tribenzoic acid (H_3_TATB), followed by in situ growth of Au nanoparticles (Au‐NPs) and post‐encapsulation of Hb. The resulting Au@MOF^Hb^‐NCs allow small‐molecule diffusion, bind and release O_2_, and exhibit antioxidant reactivity toward reactive oxygen species (ROS). (b) STEM images of (i, ii) empty MOF‐NCs and (iii, iv) Au@MOF‐NCs synthesized using 50 µL of 5.88 mm chloroauric acid (HAuCl_4_) (Au50@MOF‐NC). Insets show magnified lattice features, including the corresponding Fast Fourier Transform pattern with 6.8 nm spacing and ∼3 nm AuNPs. (c) Catalytic activity of Au@MOF‐NCs. (i) Catalase (CAT)‐like activity of Au@MOF‐NCs prepared with 10, 25, 50, and 100 µL of 5.88 mm HAuCl_4_ (Au10@MOF‐NC, Au25@MOF‐NC, Au50@MOF‐NC, and Au100@MOF‐NC). Activity was assessed using the hydrogen peroxide (H_2_O_2_)‐dependent oxidation of Amplex Red to resorufin via horseradish peroxidase (HRP), presented as normalized mean fluorescence intensity (nMFI) over time (left) and across repeated catalytic cycles (right), (ii) superoxide dismutase (SOD)‐like activity of Au@MOF‐NCs determined via WST‐1 reduction by superoxide radicals (O_2_^•−^) generated by the xanthine/xanthine oxidase (XO) system. Normalized absorbance (nAbs) is reported over time (left) and over multiple cycles (right). Adapted with permission [9]. Copyright 2023 Royal Society of Chemistry.

Our group also pioneered the integration of Au‐based nanozymes into HBOCs. In an initial study, AuNPs were incorporated into MOF‐based NCs to provide antioxidant protection and preserve Hb functionality [9]. MOFs are highly ordered, porous coordination networks with tunable pore sizes and large surface areas, making them well‐suited for biomolecule encapsulation [97, 98, 99, 100, 101, 102]. AuNPs were grown in situ within the MOF‐type porous coordination network (PCN)‐333, which had previously been shown to enable high Hb loading and oxygen‐carrying capacity (Figure 2a) [9, 103, 104]. AuNPs were selected because they represent one of the most extensively studied nanozymes for biomedical applications [105, 106]. Their synthesis is straightforward, cost‐effective, and allows precise control over morphological features (e.g., size and shape), which directly influence catalytic performance and biocompatibility [107]. Importantly, AuNPs exhibit multiple enzyme‐mimicking activities, particularly CAT‐ and SOD‐like functions. The resulting AuNPs@PCN‐333 NCs displayed uniform size (∼200 nm, polydispersity index < 0.2) (Figure 2b (i, ii)). and well‐dispersed AuNPs (∼3 nm) within the MOF cages (∼6.8 nm) (Figure 2b (iii, iv)). Catalytically, the resulting AuNPs@PCN‐333 NCs decomposed ∼45% of H_2_O_2_ in the first reaction cycle and retained ∼25% of activity after five cycles (Figure 2c (i)). Likewise, O_2_^•^^−^ scavenging efficiency reached 60%, with ∼40% of activity maintained upon repeated use, demonstrating good recyclability and stability (Figure 2c (ii)). Post‐encapsulation of bovine Hb yielded AuNPs@PCN‐333‐Hb NCs that largely preserved Hb secondary structure and reversible oxygen binding. UV–vis spectroscopy confirmed intact oxygenation/deoxygenation behavior, while AuNP incorporation reduced metHb formation under H_2_O_2_ challenge by ∼8% compared to Au‐free controls. Importantly, the hybrids remained highly biocompatible, with RAW 264.7 and HUVEC viabilities exceeding 100% up to 125 µg mL^−1^. Together, these results demonstrate that embedding Au‐based nanozymes within MOF‐hosted Hb carriers enables multifunctional HBOCs combining efficient oxygen transport with recyclable antioxidant protection, thereby mitigating Hb autoxidation and enhancing long‐term stability.

**FIGURE 3:**
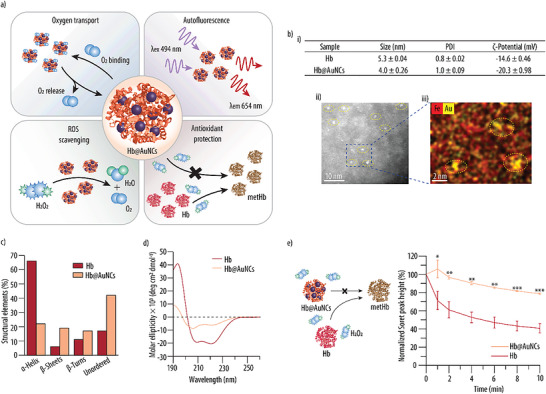
(a) Schematic illustration of hemoglobin (Hb)‐protected gold nanoclusters (NCs) (Hb@AuNCs). Hb provides reversible O_2_ binding and release, while the Au nanocluster (AuNC) component imparts autofluorescence (excitation wavelength (λ_ex_) at 494 nm and emission wavelength (λ_em_) at 654 nm) and antioxidant activity toward reactive oxygen species (ROS), including hydrogen peroxide (H_2_O_2_), thereby limiting conversion of Hb to methemoglobin (metHb). (b) (i) Hydrodynamic size, polydispersity index (PDI), and zeta (ζ)‐potential of free Hb and Hb@AuNCs, with corresponding size distribution profiles, (ii) ADF‐STEM image of Hb@AuNCs, and (iii) EDX elemental map showing the distribution of Fe (red) and Au (yellow). (c) Secondary structure analysis of free Hb and Hb@AuNCs, showing relative contributions of α‐helices, β‐sheets, β‐turns, and unordered elements. (d) Circular dichroism spectra of Hb and Hb@AuNCs, confirming structural rearrangement upon nanocluster formation. (e) Normalized Soret peak height over time for Hb and Hb@AuNCs incubated with 0.09 mm H_2_O_2_, demonstrating antioxidant properties of Hb@AuNCs. Adapted with permission [11]. Copyright 2023 Royal Society of Chemistry.

Besides AuNPs, we also investigated the use of Au nanoclusters (AuNCLs), ultrasmall aggregates of only a few to tens of Au atoms that combine enzyme‐like catalytic activity with intrinsic fluorescence (Figure 3a) [11]. Owing to their ultrasmall size, AuNCLs typically exhibit higher catalytic activity than larger NPs but suffer from poor colloidal stability due to aggregation. To overcome this limitation, we employed Hb as both a reducing and stabilizing agent to direct in situ AuNCL synthesis, yielding protein‐protected AuNCLs (Hb@AuNCLs) for the first time in the context of blood surrogates. The resulting hybrids exhibited a hydrodynamic diameter of ∼4.0 nm and a negative ζ‐potential, with uniformly distributed Au cores of 0.5–1.5 nm embedded within the Hb matrix, as confirmed by annular dark filed (ADF)‐scanning transmission electron microscopy (STEM) and energy‐dispersive X‐ray (EDX) mapping (Figure 3b (i–iii)). The Hb@AuNCLs displayed red fluorescence (λ_em_ = 654 nm, λ_exc_ = 494 nm), enabling potential in vivo tracking [108, 109]. Structural analyses revealed partial conformational rearrangement of Hb upon AuNCL formation, with reduced α‐helical content and increased β‐sheet contributions (Figure 3c), consistent with circular dichroism measurements (Figure 3d). Despite these changes, reversible oxygen binding was largely preserved, as evidenced by characteristic Soret band shifts during repeated oxygenation/deoxygenation cycles. Owing to their CAT‐like activity, Hb@AuNCLs efficiently scavenged ROS, rapidly decomposing H_2_O_2_ while retaining ∼50% catalytic activity after multiple reaction cycles. This antioxidant activity translated into pronounced protection against Hb oxidation. Upon H_2_O_2_ challenge, Hb@AuNCLs exhibited minimal Soret peak loss compared to free Hb (Figure 3e), corresponding to a ∼39% reduction in metHb formation. This protection was markedly stronger than that achieved with AuNP‐based MOF systems and comparable to that reported for Hb‐(HSA(PtNP))_3_ clusters [9, 10]. To mitigate potential in vivo concerns related to the small size of Hb@AuNCLs, the hybrids were encapsulated within PCN‐333 MOF NCs. Successful encapsulation was confirmed by shifts in ζ‐potential, increased particle size, and homogeneous distribution of Fe, Au, and Al throughout the structure, as observed by SEM, ADF‐STEM, and EDX mapping. This creative design illustrates how integrating nanocatalysts at the molecular level with Hb can yield HBOCs that are not only oxygen carriers and ROS sponges, but even theranostic agents (with imaging capability).

An early but conceptually prescient strategy to endow HBOCs with intrinsic antioxidant functionality was reported by Shellington et al., who developed a polynitroxylated PEGylated Hb (PNPH) formulation designed to mitigate oxidative and NO‐related toxicity while preserving oxygen delivery. In this system, bovine Hb was covalently modified with both PEG chains and approximately fourteen nitroxide radicals per Hb tetramer [110]. Nitroxides are stable free radicals with well‐established SOD‐mimetic activity and, through redox coupling with the heme iron, additional CAT‐like behavior, thereby enabling the catalytic detoxification of ROS. PEGylation further increased the hydrodynamic size of the HBOC, reduced endothelial interactions and NO scavenging, and imparted favorable colloidal and oncotic properties. Importantly, in a murine model of traumatic brain injury combined with hemorrhagic hypotension, PNPH enabled small‐volume resuscitation, restored mean arterial pressure more efficiently than lactated Ringer's or Hextend (an isotonic crystalloid and colloid solution, respectively, widely used for volume replacement in trauma and surgical settings), and significantly reduced neuronal degeneration in the hippocampal CA1 region at 7 days post‐injury. These effects were attributed to the combined oxygen‐carrying capacity and catalytic ROS‐scavenging activity of the nitroxide‐decorated Hb, highlighting the therapeutic potential of integrating stable, enzyme‐mimicking redox motifs directly into HBOCs. Although PNPH relies on covalently attached molecular antioxidants rather than inorganic nanomaterials, this work anticipated many of the core principles underpinning modern nanozyme‐integrated HBOCs, namely the use of robust, catalytic, non‐biological mimics of erythrocytic antioxidant defenses to enhance safety and functional performance under oxidative stress.

Beyond oxygen delivery, native RBCs play a crucial role in maintaining systemic acid‐base homeostasis through the carbonic anhydrase (CA)‐mediated hydration of carbon dioxide (CO_2_) into bicarbonate (HCO_3_^−^) [111, 112]. This reaction enables efficient CO_2_ clearance and buffering of blood pH during gas exchange. However, nearly all reported HBOCs focus exclusively on oxygen transport, overlooking this equally vital function. The integration of CA‐like activity into HBOC systems would thus represent an important step toward reproducing the full gas‐exchange functionality of native RBCs, particularly under conditions of acidosis or hypoventilation. In this context, Pablo‐Sainz‐Ezquerra et al. recently introduced Hb‐encapsulating zeolitic imidazolate framework (ZIF)‐8 NPs (Hb@ZIF‐8 NPs) that combine oxygen transport with CA‐mimetic activity within a single nanoplatform [113]. The Zn^2+^ centers of ZIF‐8, tetrahedrally coordinated by imidazolate ligands, mimic the catalytic motif of native CA (Zn^2+^ ion coordinated by three histidine residues and one water molecule), enabling the reversible hydration of CO_2_ in addition to stabilizing the encapsulated Hb. The resulting Hb@ZIF‐8 NPs displayed pronounced Michaelis‐Menten behavior in the hydrolysis of *p*‐nitrophenyl acetate (R^2^ > 0.99), confirming true enzyme‐like catalysis. Spectroscopic analysis confirmed that encapsulated Hb preserved its reversible oxygen‐binding capacity, as evidenced by the characteristic Soret band shift from 413 to 429 nm upon deoxygenation and its return to 413 nm upon reoxygenation.

In summary, the integration of nanozymes and enzyme‐mimetic motifs into HBOCs offers a powerful strategy to address one of the field's most persistent challenges: the oxidative degradation of Hb outside the RBC. By partially recapitulating the endogenous antioxidant enzymatic defenses of erythrocytes, these advanced HBOCs combine efficient oxygen transport with robust, long‐lasting antioxidant protection. From early redox‐active designs such as polynitroxylated PEGylated Hb, which demonstrated in vivo neuroprotection and improved hemodynamic performance in a traumatic brain injury and hemorrhagic hypotension model, to pioneering PtNP‐HSA‐Hb clusters and more recent CeO_2_‐ and Au‐based systems, these hybrid constructs collectively demonstrate that catalytic mimics can effectively preserve Hb functionality, mitigate ROS‐induced damage, and even add multifunctionality such as fluorescence for imaging or membrane coatings for immune evasion. Notably, CA‐mimicking frameworks such as Hb@ZIF‐8 NPs extend biomimicry beyond oxygen delivery, opening the possibility of CO_2_ hydration and pH buffering analogous to native RBCs. Collectively, these advances establish a strong conceptual and technological foundation for next‐generation artificial oxygen carriers. However, important challenges still remain to be addressed. ROS‐scavenging activity is often assessed using simplified in vitro assays that do not fully capture the complexity of redox regulation in vivo. Physiological redox homeostasis arises from dynamic processes involving ROS flux, compartmentalization, oxygen gradients, and interactions with endogenous antioxidant enzymes. Bridging the gap requires more rigorous evaluation frameworks, including assessments of catalytic durability under repeated ROS challenge, oxygen–ROS coupling, biological competition, and cellular redox buffering. Assays incorporating multiple ROS exposure cycles, complex biological matrices, oxygen gradients, and intracellular redox readouts (e.g., reduced and oxidized glutathione balance) offer more physiologically meaningful insight than single‐endpoint scavenging tests. Finally, although comprehensive in vivo validation, including large‐volume blood replacement studies, remains limited, nanozyme‐integrated HBOCs should be viewed as early‐stage yet highly promising candidates for advanced blood substitutes.

## 4 Nanozyme‐Integrated HBOCs for Wound Healing

Wound healing is a dynamic, multistage process encompassing inflammation, proliferation, and remodeling, each requiring an adequate oxygen supply to sustain cellular metabolism and extracellular matrix (ECM) synthesis [114]. Oxygen is not only a metabolic substrate but also a key signaling molecule that regulates skin cell proliferation, granulation, re‐epithelialization, angiogenesis, and tissue regeneration [115]. Physiological oxygen tension in healthy skin typically ranges between 35 and 50 mmHg, while in chronic wounds it often falls below 10 mmHg, creating a hypoxic microenvironment that severely impairs cellular activity and delays healing [116, 117]. Prolonged hypoxia further promotes inflammation and predisposes the wound bed to bacterial infection, establishing a harmful cycle that perpetuates non‐healing states [116, 117]. Chronic wounds, particularly diabetic ulcers, venous leg ulcers, and pressure sores, are therefore characterized by microcirculatory dysfunction, excessive oxidative stress, and impaired angiogenic signaling, all of which are strongly oxygen‐dependent [117].

Conventional oxygen therapies, such as hyperbaric oxygen therapy (HBOT) and topical oxygen therapy (TOT), can elevate local oxygen but generally fail to sustain physiological gradients or deliver oxygen with spatiotemporal precision [117]. In the case of HBOT, tissue reoxygenation relies on an intact vascular system. Although HBOT increases blood partial oxygen pressure (pO_2_), oxygen can only reach the wound site through blood circulation, an issue in many chronic wounds, which are characterized by vascular insufficiency. Retrospective studies have reported that HBOT improves wound healing in the short term. However, its long‐term efficacy remains uncertain and inconsistent across clinical evaluations [117, 118]. TOT, by contrast, administers pure oxygen directly to the wound, typically via a portable inflatable chamber [119]. This bypass microcirculatory dependence and offers pragmatic advantages: lower cost, reduced risk of oxygen toxicity, and home‐based convenience [117, 119]. Still, both HBOT and TOT are largely passive since, once applied, oxygen diffuses according to local gradients and cannot be tuned dynamically to match the wound's evolving metabolic demands. Neither approach, on its own, addresses the array of co‐limiting factors (i.e., bacterial burden, persistent inflammation, redox imbalance) that characterize chronic wounds.

HBOCs aim to bridge this gap by providing a biomimetic route for delivering molecular oxygen directly to hypoxic tissues, decoupled from systemic perfusion. By exploiting the reversible oxygen‐binding properties of Hb, HBOCs can serve as injectable or implantable “oxygen depots” to restore normoxia within the wound microenvironment. Nevertheless, the therapeutic efficacy of traditional HBOCs remains constrained by uncontrolled oxygen‐release kinetics and lack of multifunctionality: excessive oxygen can amplify oxidative damage, whereas insufficient release renders the system ineffective [12, 114]. Moreover, chronic wounds are not solely governed by hypoxia since microbial infection, inflammation, and redox imbalance must also be addressed simultaneously to achieve full tissue recovery.

**SCHEME 3:**
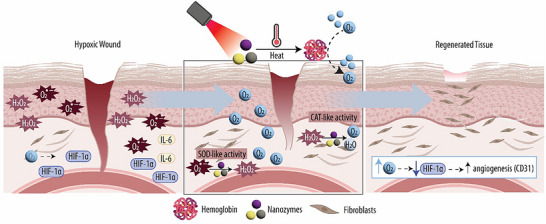
Schematic illustration of the therapeutic mechanism of hemoglobin (Hb)–nanozyme platforms for the treatment of hypoxic wounds. Hypoxic wound environments are characterized by limited oxygen availability and elevated levels of reactive oxygen species (ROS), including superoxide (O_2_^•−^) and hydrogen peroxide (H_2_O_2_), which stabilize hypoxia‐inducible factor‐1α (HIF‐1α) and promote inflammatory signaling (e.g., interleukin‐6 (IL‐6)), thereby impairing tissue repair. Upon near‐infrared triggered heating, the hybrid nanozyme–HBOC platform is activated. Photothermal heating triggers O_2_ release from Hb, while nanozymes catalytically regulate ROS through superoxide dismutase (SOD)‐like activity (2O_2_^•−^ + 2H^+^ → H_2_O_2_ + O_2_) and catalase (CAT)‐like activity (2H_2_O_2_→ 2H_2_O + O_2_), thereby restoring redox homeostasis (center). The combined oxygen supplementation and ROS scavenging alleviate hypoxia, downregulate HIF‐1α signaling, promote angiogenesis (e.g., increased cluster of differentiation 31 (CD31) expression), and support fibroblast proliferation, ultimately leading to accelerated tissue regeneration and wound healing. Made and modified from BioRender.

Recent research therefore turns to hybrid platforms that pair HBOCs with nanozymes to create intelligent, externally controllable therapies. By combining Hb's oxygen‐carrying capacity with the catalytic and/or photothermal functions of nanozymes, these systems enable spatiotemporally precise oxygen delivery and add secondary activities such as ROS modulation, antibacterial catalysis, or near‐infrared (NIR)‐triggered heating [12]. When embedded in biocompatible matrices, nanozyme‐integrated HBOC not only re‐oxygenate tissue but also promote angiogenesis, collagen maturation, and epithelial regeneration. Collectively, they represent a new generation of “intelligent” oxygen carriers that move beyond passive delivery toward bioresponsive, multipronged wound therapy (Scheme 3).

**FIGURE 4:**
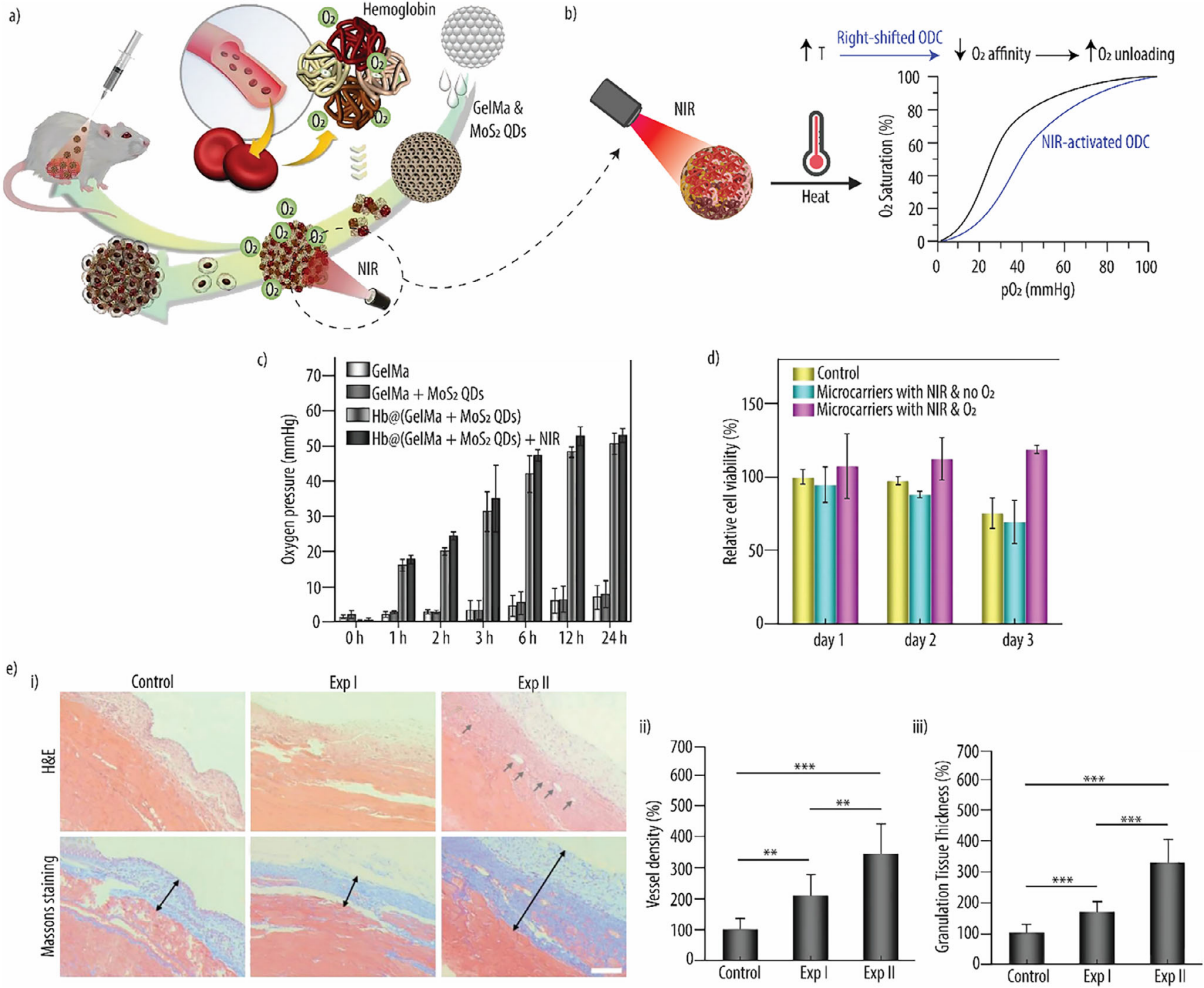
(a) Schematic illustration of the molybdenum disulfide (MoS_2_) quantum dot (QD)‐gelatin methacryloyl (GelMa) inverse‐opal microcarrier, in which hemoglobin (Hb) is covalently anchored to a GelMa scaffold templated by monodisperse silica colloidal crystal beads that supports controlled O_2_ diffusion and near‐infrared (NIR) photothermal responsiveness. (b) Mechanistic illustration of NIR‐triggered photothermal modulation of Hb O_2_ binding, in which local heating induced by MoS_2_ QDs shifts the O_2_ dissociation curve (ODC) rightward, decreasing Hb–O_2_ affinity and thereby enhancing O_2_ unloading at a given partial pressure of O_2_ (pO_2_). (c) O_2_‐release behavior of four variants: plain GelMa, GelMa containing MoS_2_ QDs, GelMa containing both MoS_2_ QDs and Hb, and the Hb/MoS_2_ QD system irradiated with NIR irradiation. (d) Relative cell viability of NIH 3T3 fibroblasts cultured with the microcarriers under hypoxia, quantified by MTT assay. (e) (i) Histological assessment of tissue repair in a Sprague‐Dawley rat abdominal wall defect model 2 weeks post‐treatment, showing hematoxylin and eosin (H&E) staining of control, experimental I (Exp I), and experimental II (Exp II) groups (vessels indicated by arrowheads), and Masson's trichrome staining highlighting granulation tissue thickness (arrowheads). Scale bar: 200 µm. Quantitative analysis of (ii) vessel density and (iii) granulation tissue thickness. **p < 0.01, ***p < 0.001. Adapted with permission [12]. Copyright 2019 John Wiley and Sons.

A representative platform is the molybdenum disulfide (MoS_2_) quantum dots (QDs) and gelatin methacryloyl (GelMa) inverse‐opal microcarrier reported by Liu et al. [12]. In this design, Hb is covalently anchored to a GelMa scaffold structured as an inverse opal using monodisperse silica colloidal crystal beads as sacrificial templates (Figure 4a). The resulting architecture presents a well‐ordered, interconnected porous network with nanochannels that facilitate oxygen diffusion, allowing Hb to unload oxygen efficiently into the surrounding tissue. To regulate both the timing and magnitude of oxygen release, MoS_2_ QDs are incorporated to impart light responsiveness: upon NIR exposure, MoS_2_ converts light to heat, shifting Hb's oxygen dissociation curve (ODC) to favor desaturation (Figure 4b) [114, 120, 121]. This photothermal lever is central to spatiotemporal control: by modulating irradiation parameters (power density, duration, duty cycle), oxygen desaturation can be increased or paused on demand without altering the scaffold's composition.

MoS_2_ is a 2D transition metal dichalcogenide with strong optical absorption in the NIR, favorable electronic properties, large surface area, and good biocompatibility and stability [122, 123, 124]. Its robust photothermal effect supports temperature‐controlled release of biomolecules [125, 126], and the QD form (<10 nm lateral size) retains these advantages while enhancing dispersion and biomedical compatibility [127, 128]. Inverse opals, on the other hand, are highly ordered porous structures with large surface areas and interconnected nanochannels that facilitate molecule loading, cell adhesion, and monitoring via their structural color [129, 130, 131, 132]. GelMa adds ECM‐mimetic biochemical cues and tunable mechanics, supporting cell adhesion, proliferation, and tissue ingrowth while allowing straightforward chemical conjugation to Hb [133, 134]. Mechanistically, the ODC shifts rightward with increasing temperature, reducing Hb's affinity for oxygen and promoting its release [135]. Thus, MoS_2_’s photothermal response provides a gentle, remote trigger to modulate oxygenation within a clinically relevant range.

The microcarriers were fabricated using monodisperse silica colloidal crystal beads generated by microfluidic single‐emulsion, yielding a narrow size distribution and ensuring uniform pore replication after template removal. MoS_2_ QDs of <10 nm lateral size are embedded during GelMa curing, and Hb is coupled through EDC/NHS chemistry. Oxygen‐release studies benchmark four variants: plain GelMa, GelMa with MoS_2_ QDs, GelMa containing both MoS_2_ QDs and Hb, and the latter exposed to NIR light. After 24 h in hypoxic medium, only Hb‐loaded microcarriers showed a marked rise in dissolved oxygen tension from ∼7–8 mmHg to ∼50 mmHg (Figure 4c). With NIR irradiation, release is faster and reaches higher plateaus due to photothermal acceleration of Hb desaturation. Importantly, the system is not merely an oxygen “leak” since the MoS_2_ trigger allows pulses or cycles of release adapted to need.

Cytocompatibility and function are evaluated with NIH 3T3 fibroblasts cultured under hypoxia. Only the oxygen‐releasing Hb/MoS_2_ QDs microcarriers under NIR maintain normal cell morphology and proliferation over 3 days, while cells on oxygen‐inert controls exhibit reduced metabolic activity. MTT assay confirms significantly higher viability for the photoactivated oxygen group, and NIR alone shows no cytotoxicity under the applied parameters providing evidence that the photothermal window is appropriately mild (Figure 4d). In vivo, in Sprague–Dawley rats bearing 1 × 1 cm partial abdominal wall defect, histology after two weeks reveals minimal inflammation across groups, indicating good biocompatibility. However, only the oxygen‐loaded, NIR‐activated microcarriers markedly enhance regeneration, with significantly denser collagen by Masson's staining (Figure 4e (i)), greater vessel density (Figure 4e (ii)), and thicker granulation (Figure 4e (iii)). Altogether, the platform elevates local oxygen from severe hypoxia (∼7 mmHg) to near‐physiological levels (∼50 mmHg) within 24 h, translating into improved angiogenesis and ECM deposition. The take‐home message is clear: combining structural porosity, Hb cargo, and MoS_2_ QDs photothermal control yields a tunable, on‐demand oxygen reservoir that supports tissue repair.

**FIGURE 5:**
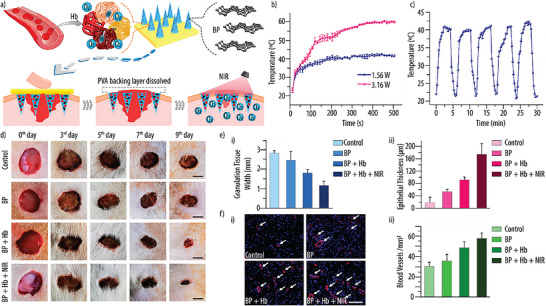
(a) Schematic illustration of black phosphorus (BP) quantum dot (QD)‐hemoglobin (Hb) microneedles (MNs) designed for transdermal, near‐infrared (NIR)‐responsive O_2_ delivery. The MNs consist of a dissolvable poly(vinyl acetate) (PVA) backing and GelMA tips co‐encapsulating BP QDs as the photothermal transducer and Hb as the O_2_ source, enabling controlled Hb desaturation upon gentle NIR irradiation. (b) Temperature‐rising profiles of MNs under two NIR power densities (1.56 and 3.16 W), demonstrating tunable photothermal response. (c) Reversible and repeatable temperature modulation of MNs over five on/off irradiation cycles. (d) Wound‐healing performance in a type I diabetic rat model with 1 cm full‐thickness wounds, showing representative photographs of the control, BP, BP + Hb, and BP + Hb + NIR groups on days 0, 3, 5, 7, and 9. Scale bars: 0.5 cm. (e) Quantitative analysis of day‐9 (i) granulation tissue width and (ii) epithelial thickness across groups. (f) (i) Mechanistic evaluation of healing on day 9 using Masson's trichrome staining and a platelet endothelial cell adhesion molecule‐1/alpha–smooth muscle actin double immunofluorescent staining, with vascular structures indicated by arrows. Scale bar: 100 µm. (ii) Quantitative analysis of vessel density on day 9 is also shown. Adapted with permission [114]. Copyright 2020 American Chemical Society.

Transdermal strategies push this concept further by integrating controllable oxygen release directly into a minimally invasive delivery device. Zhang et al. introduced black phosphorous (BP) QDs–Hb microneedles (MNs) that merged photothermal nanozyme behavior with direct intradermal oxygen release [114]. MNs can painlessly and safely penetrate the skin to reach underlying tissues, enabling efficient transdermal delivery of various therapeutic agents, including small molecules, proteins, and cytokines [136, 137, 138, 139, 140]. Meanwhile, BP, a 2D nanomaterial with strong NIR absorption, high light‐to‐heat conversion efficiency, and favorable biodegradation and biocompatibility profiles [141, 142, 143, 144], acts as the photothermal switch. In this design, the MNs consisted of a poly(vinyl acetate) backing layer that rapidly dissolved within 10 min upon skin insertion, leaving biocompatible GelMA tips embedded inside the tissue (Figure 5a). These tips co‐encapsulate Hb as the oxygen source and BP QDs as the photothermal transducer, so that gentle NIR pulses elevate local temperature within safe limits and trigger Hb desaturation.

Temperature control is central to safety and efficacy. In the reported system, 3.16 W NIR elevates MN temperature to ∼60°C within 2 min, while 1.56 W achieves ∼40°C within 3 min (Figure 5b). The lower setting is typically sufficient to trigger oxygen release without thermal damage, heating is reversible and repeatable over multiple cycles, indicating that the photothermal response is stable under on/off control (Figure 5c). In hypoxic media, Hb‐containing MNs increase dissolved oxygen relative to controls, and NIR accelerates and amplifies this release, demonstrating precise photothermal regulation.

Therapeutically, in a type I diabetic rat model with 1 cm full‐thickness wounds, BP QDs‐Hb MNs under NIR achieve near‐complete closure by day 9 (Figure 5d). Hematoxylin‐eosin staining shows faster contraction and thicker epithelium and quantitative metrics confirm smallest wound areas and narrowest beds, as shown in Figure 5e (i), and the BP QDs + Hb + NIR group showed the thickest epithelial layers (Figure 5e (ii)). Mechanistic staining supports these observed outcomes. As presented in Figure 5f (i), Masson's reveals dense, aligned collagen and inteurleukin (IL)‐6 expression is reduced, indicating reduced infection or irritation while a platelet endothelial cell adhesion molecule‐1 (CD31)/alpha–smooth muscle actin (α‐SMA) double staining revealed the highest blood vessel density in the BP QDs + Hb + NIR group, consistent with oxygen‐driven vascular regeneration (Figure 5f (ii)). Although NIR alone confers minor healing benefits probably via improved microcirculation and low‐level ROS signaling, combining BP QDs‐mediated photothermal activation with Hb oxygen delivery produced superior outcomes. Mechanistically, mild photothermal activation of BP nanozymes modulates multiple wound‐healing pathways simultaneously: i) thermally triggered Hb oxygen release mitigates hypoxia and reduces hypoxia‐inducible factor 1α (HIF‐1α) expression; ii) localized heating enhances fibroblast proliferation and ECM deposition; and iii) the photothermal effect assists in bacterial suppression by transiently elevating temperature and generating low levels of ROS (Scheme 3). Thus, BP nanozymes act not only as thermal transducers but also as catalytic mediators that integrate energy conversion and biochemical oxygen regulation. The resulting MN system demonstrates how combining a photothermal nanozyme with an HBOC transforms a passive oxygen source into an intelligent, light‐controllable wound‐healing platform.

Beyond hydrogels and MNs, electrospun nanofibers enable large‐area, conformal wound dressings with high surface area and ECM‐like architecture. Zhao et al. reported a multifunctional electrospun dressing in which electrospun poly‐*L*‐lactic acid (PLLA) and quaternized chitosan (QCS) nanofibers were alternately coated with negatively charged hyaluronic acid (HA) layers containing Hb and BP nanosheets, forming an n‐layered PLLA/QCS/BP/Hb‐HA nanofiber construct (PQBH‐n), where “n” denotes the number of layers (0, 2, 4, or 8) [145, 146, 147]. This design integrates oxygen release, antibacterial action, and hemostasis, which is especially relevant to diabetic skin injury, where microvasculature, immune response, and redox balance are perturbed. Here, Hb provided thermally responsive oxygenation [120, 121], BP supplied NIR‐triggered photothermal control [148], and QCS contributed intrinsic antibacterial and hemostatic activity [149, 150, 151].

The nanofibers properties are tuned intelligently: as layers increase, hydrophilicity improves (contact angle drops from 121° for bare PLLA to 73° for an eight‐layered PLLA/QCS/BP/Hb‐HA nanofiber construct (PQBH‐8)), favoring cell adhesion and potentially also wound healing [152]. BP degradation products are non‐toxic toward fibroblasts and endothelial cells, and NIR exposure heats films to 52.8°C in vitro and ∼40°C on skin, which is a safe, effective range for triggering Hb‐mediated oxygen release in an on/off manner. Antibacterial performance is striking: under NIR, PQBH‐8 achieves 98.2% inhibition against methicillin‐resistant *Staphylococcus aureus* (MRSA) and 90.4% against *Escherichia coli* (E. coli), reflecting synergy between QCS and BP. QCS disrupts bacterial membranes through electrostatic and hydrophobic interactions, while BP physically damages cell walls and, upon NIR exposure, generates local heat and ROS that enhance bacterial killing [153, 154, 155, 156].

In a diabetic‐mimic culture (1% O_2_, 33 mm glucose), PQBH‐8 + NIR reduces HIF‐1α expression, confirming effective reoxygenation, and significantly enhances fibroblast and endothelial proliferation and migration (L929 fibroblast cell line: 83%; HUVECs: 72%). Capillary‐like networks become longer and more branched, indicating enhanced angiogenic potential, which is critical for re‐establishing sustained oxygen supply [157]. In streptozotocin‐induced diabetic mice, PQBH‐8 + NIR accelerates wound closure and reduces scarring (scar elevation index 0.49 vs. 1.02 for untreated controls). Histology reveals thicker epithelium, denser collagen with a higher type III/I ratio, abundant hair follicles, increased Ki‐67 (proliferation), and reduced caspase‐3 (apoptosis). Pro‐angiogenic markers (CD31, α‐SMA, vascular endothelial growth factor) increase while HIF‐1α decreases, consistent with oxygen normalization. α‐SMA is an important indicator of smooth muscle and mature blood vessels [158]. CD31 is a transmembrane protein that plays a vital role in early angiogenesis [159]. Inflammatory cytokines (IL‐6, IL‐1β, tumor necrosis factor‐alpha) are suppressed while anti‐inflammatory markers (IL‐10, IL‐13) and M2 polarization (CD206^+^ ∼15.5% ± 0.9%) are enhanced, indicating a shift toward a pro‐regenerative immune milieu. Together, the dressing unifies oxygenation, antibacterial defense, anti‐inflammatory regulation, and vascular regeneration in a single, NIR‐responsive construct tailored to the harsh biology of diabetic wounds.

Taken together, these platforms highlight a convergent design logic. First, precise oxygen control is indispensable: MoS_2_ and BP QDs or BP nanosheets provide gentle, NIR‐tunable heating that right‐shifts the ODC, enabling on‐demand Hb desaturation without systemic exposure or uncontrolled diffusion. Second, multifunctionality matters: catalysis (peroxidase‐like activity), photothermal action, and immunomodulation address infection, inflammation, and redox imbalance alongside hypoxia. Third, tissue integration is key: GelMa hydrogels, inverse‐opal scaffolds, MN tips, and electrospun fibers offer ECM‐like mechanics, high surface area, and conformal contact that support cell adhesion, vascular infiltration, and epithelial closure. Fourth, safety windows are achievable: the reported NIR parameters remain within ranges that avoid thermal damage yet deliver sufficient heating to trigger oxygen release and catalysis, and the materials exhibit good cytocompatibility and acceptable biodegradation profiles in vitro and in vivo.

From a translational perspective, the field is moving from passive HBOCs toward intelligent, stimuli‐responsive wound‐healing platforms. Inverse‐opal carriers suggest scalable microfluidic templating routes with uniform pore architectures; MN systems offer minimally invasive, patient‐friendly delivery with precise dosing through pulse protocols; electrospun dressings allow large‐area coverage and straightforward application in outpatient settings. Each strategy can be tailored to wound type: for example, MNs for localized, deep dermal lesions; electrospun dressings for broad, superficial diabetic ulcers; and injectable microcarriers for irregular geometries or deep pockets. The ability to “dial in” oxygen delivery, either continuously at low levels to prevent hypoxia rebound, or in bursts to match peaks in metabolic demand, could be critical to optimizing outcomes while minimizing oxidative stress. Layered on top of oxygenation, the catalytic and photothermal features of nanozymes create a modular toolbox to suppress infection, dampen maladaptive inflammation, and stimulate angiogenesis, shortening time‐to‐closure and improving scar quality.

## 5 Applications for Antitumor Therapy

**SCHEME 4:**
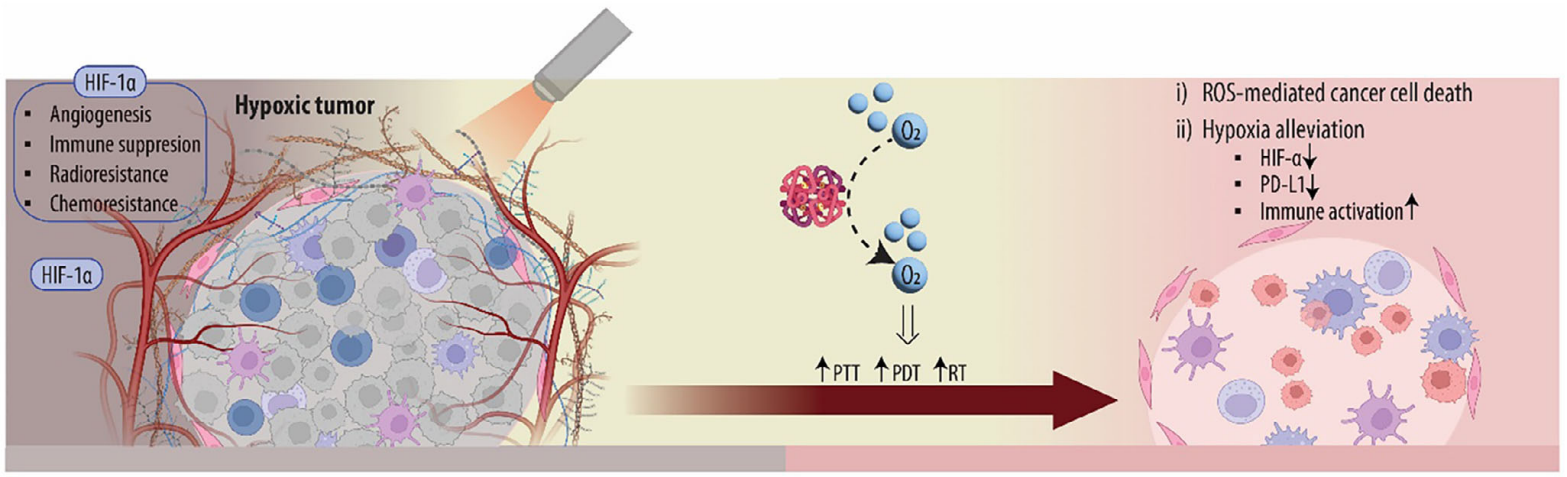
Schematic illustration of the therapeutic role of hemoglobin (Hb)–nanozyme systems in hypoxic tumors. The tumor microenvironment is characterized by severe oxygen depletion and stabilization of hypoxia‐inducible factor‐1α (HIF‐1α), which promotes angiogenesis, immune suppression, and resistance to radiotherapy and chemotherapy. Upon administration, Hb‐based oxygen carriers deliver molecular oxygen (O_2_) to the tumor tissue, alleviating hypoxia. In parallel, nanozymes modulate reactive oxygen species (ROS) levels, enabling ROS‐mediated cancer cell damage while simultaneously improving oxygen availability. The resulting increase in intratumoral O_2_ enhances the efficacy of oxygen‐dependent therapies such as photodynamic therapy (PDT), photothermal therapy (PTT), and radiotherapy (RT). Relief of hypoxia downregulates HIF‐1α and programmed death‐ligand 1 (PD‐L1) expression and promotes immune activation, ultimately leading to improved antitumor responses and cancer cell death.

Tumor hypoxia remains a formidable barrier to effective cancer therapy. Solid tumors frequently display regions of low oxygen tension due to abnormally structured vasculature, heterogeneous and inefficient perfusion, elevated consumption of oxygen by rapidly dividing tumor cells, and high interstitial pressure that limits diffusion of oxygen carriers [160]. Hypoxia stabilizes HIF‐1α and triggers adaptive responses including angiogenesis, epithelial‐mesenchymal transition, immune suppression, and radio‐/chemo‐resistance [161]. Because many modalities such as RT and PDT are highly dependent on molecular oxygen to generate cytotoxic ROS (e.g., ^•^OH, singlet oxygen (^1^O_2_)), their efficacy is severely compromised in hypoxic microenvironments [162, 163]. As a result, therapeutic strategies that can simultaneously alleviate hypoxia and amplify oxidative stress have attracted increasing attention (Scheme 4).

In this context, the concept of nanozyme‐assisted HBOCs has emerged as a promising approach. By combining catalytic nanomaterials, such as nanozymes that mimic enzyme functions with Hb's natural oxygen binding and release capability, one obtains hybrid systems that i) locally reoxygenate hypoxic tumor regions and ii) catalytically generate or amplify ROS, thereby synergistically undermining tumor survival [164, 165, 166]. Importantly, the source and mode of ROS generation can be externally triggered (e.g., radiation or light) or driven by tumor‐intrinsic redox cues, allowing these systems to be adapted to multiple therapeutic modalities.

**FIGURE 6:**
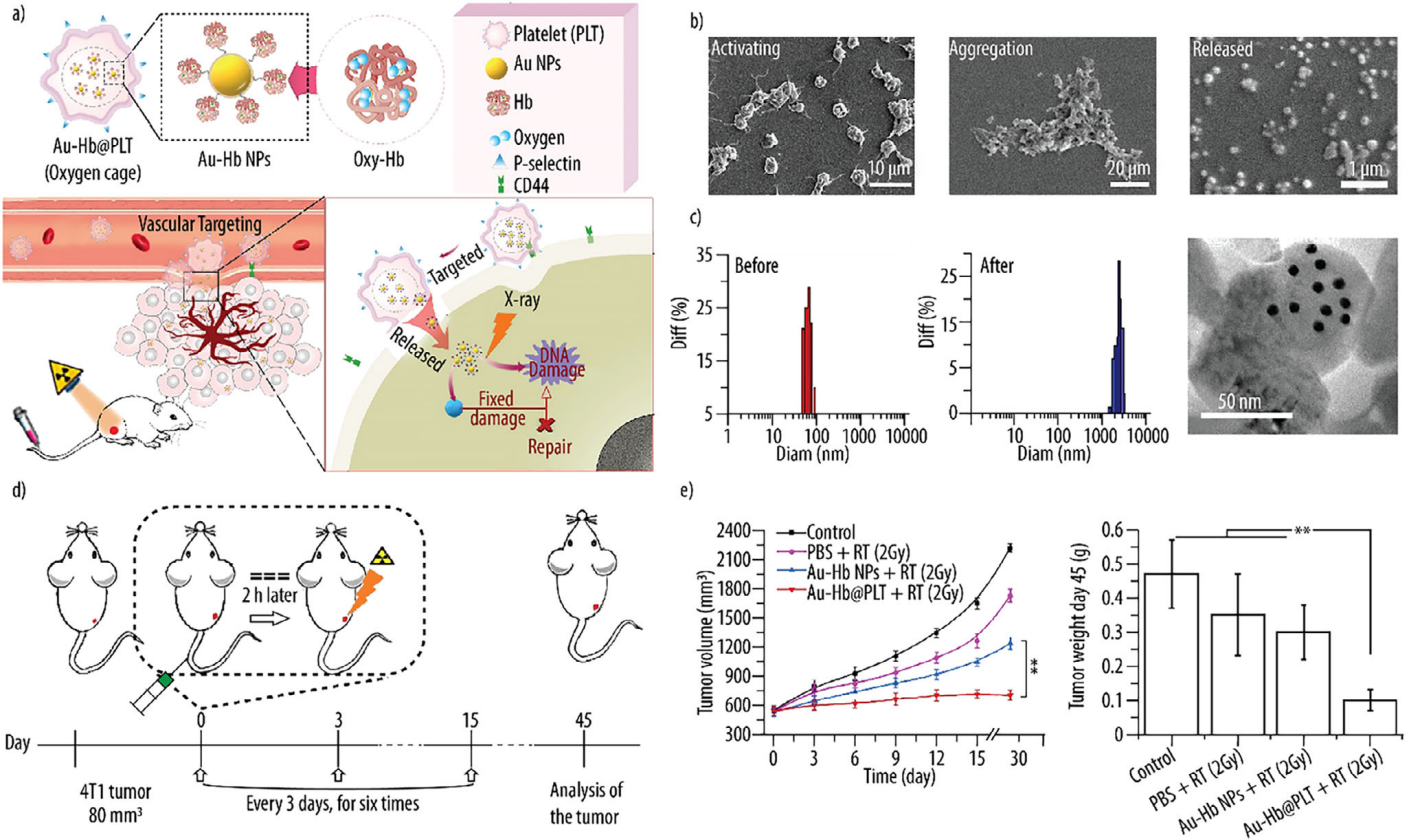
(a) Schematic illustration of the biomimetic platform in which a thiol‐modified hemoglobin (Oxy‐Hb) coordinated to Au nanoparticles (Au NPs) and subsequently encapsulated within natural platelets (PLT) (Au‐Hb@PLT). The resulting Au‐Hb@PLT system provides tumor‐homing capability, relieves tumor hypoxia, and amplifies radiation‐induced oxidative damage under X‐ray irradiation. (b) SEM micrographs showing Au‐Hb@PLT in the initial activation state, during activation, and after adenosine diphosphate (ADP)‐triggered platelet disassembly. (c) Dynamic light scattering measurements of hydrodynamic size before and after ADP activation, alongside TEM micrographs of the released platelet‐derived microparticles containing Au‐Hb NPs. (d) Schematic of the in vivo treatment regimen in HeLa tumor‐bearing mice: tumors received 2 Gy X‐ray radiotherapy (RT) every 3 days for 15 days (six cycles total), administered 2 h after intravenous injection of Au‐Hb@PLT. (e) Tumor‐growth curves (volume over 28 days) and terminal tumor weights (day 45) for control, PBS + RT (2 Gy), Au‐Hb NPs + RT (2 Gy), and Au‐Hb@PLT + RT (2 Gy) groups. Adapted with permission [14]. Copyright 2020 American Chemical Society.

In an elegant example of externally activated oxygen–ROS coupling, Xia et al. developed a biomimetic platform integrating nanozyme catalysis and oxygen delivery to enhance RT response [14]. They employed thiol‐modified Hb to anchor AuNPs via Au‐S coordination, forming Au‐HbNPs (∼18 nm). AuNPs act as redox‐active nanozymes: under X‐ray irradiation, they catalyze ROS generation (via radiation‐induced secondary electrons, enhanced local dose deposition, and possibly catalytic conversion of H_2_O_2_), thereby amplifying radiation‐induced oxidative damage (Figure 6a) [167, 168, 169, 170, 171]. Meanwhile, Hb provided oxygen transport and release, effectively reoxygenating the tumor microenvironment (TME) and sustaining ROS formation during irradiation [172, 173]. To provide targetability and immune evasion, the Au‐HbNPs were encapsulated into natural platelets, yielding Au‐Hb complex NP‐loaded platelets (Au‐Hb@PLT) (∼1.3 µm) that retained surface P‐selectin [66, 174, 175]. The platelet membrane affords i) a stealth, biocompatible shell that shields Hb from premature oxidation or oxygen leakage (via the so‐called “oxygen vault” effect), and ii) active tumor‐homing capability via P‐selectin binding to CD44 receptors on tumor cells or damaged endothelium, promoting selective accumulation at irradiated vascular sites [176, 177]. In the TME, activation by tumor‐secreted adenosine diphosphate induces platelet disassembly into smaller particles (70–100 nm) that infiltrate deeper tumor tissue (Figure 6b) [178]. Transmission electron microscopy and dynamic light scattering confirmed that released platelet‐derived microparticles contained Au‐HbNPs, thereby delivering both the oxygen reservoir and the catalytic core into hypoxic zones (Figure 6c). In vivo fluorescence imaging in HeLa tumor‐bearing mice, a model of human cervical cancer, showed markedly superior tumor accumulation of Au‐Hb@PLT compared to free Au‐HbNPs, while positron emission tomography using the hypoxia tracer ^18^F‐fluoromisonidazole (^18^F‐FMISO) confirmed effective reoxygenation in the Au‐Hb@PLT group (near‐zero ^18^F‐FMISO uptake) versus persistent hypoxia in PBS or Au‐HbNP groups. When combined with RT (2 Gy cycles), Au‐Hb@PLT achieved impressive tumor regression and even complete remission in several mice after six cycles, whereas control groups exhibited only modest growth delay (Figure 6d,e). These results underscore the synergistic feedback loop inherent to nanozyme‐integrated HBOCs: oxygen delivery by Hb enhances ROS generation by the Au nanozyme under irradiation, and the resulting oxidative stress amplifies radiation‐induced tumor damage, while the platelet cloak ensures selective delivery and extends circulation time. Thus, the Au‐Hb@PLT system exemplifies a bioinspired nanozyme‐integrated HBOC that marries oxygen transport and ROS catalysis within a single construct, illustrating how orchestrated oxygen‐ROS coupling can transform hypoxic tumors into oxidatively vulnerable targets.

Beyond externally triggered radiotherapeutic or photonic modalities, Hb‐based nano‐RBC systems have been leveraged to reprogram hypoxic tumors through endogenous, immune‐mediated mechanisms that couple oxygen delivery with selective depletion of immunosuppressive cells. In a landmark study, Wang et al. engineered a biomimetic nano RBC (nano‐RBC) platform based on amphiphilic Hb–poly(ε‐caprolactone) (Hb–PCL) conjugates that self‐assemble into hollow vesicles (V(Hb)) capable of simultaneous oxygen transport and drug delivery [179]. Distinct from membrane‐cloaked systems requiring external activation, this construct exploits the native Hb–haptoglobin (Hp) scavenging axis to achieve endogenous targeting of CD163^+^ M2‐type tumor‐associated macrophages (TAMs). Following systemic administration, doxorubicin‐loaded V(Hb) binds circulating Hp and preferentially accumulates within TAM‐rich hypoxic tumor regions, where acidic intracellular conditions trigger vesicle disassembly and doxorubicin release. In parallel, oxygen liberated from Hb alleviates local hypoxia, resulting in coordinated downregulation of HIF‐1α and programmed death‐ligand 1 (PD‐L1), suppression of IL‐10 and transforming growth factor‐beta (TGF‐β), and elevation of interferon‐gamma (IFN‐γ). In contrast to RT‐ or PDT‐oriented HBOCs, oxygen delivery here functions primarily to dismantle hypoxia‐driven immune suppression rather than to amplify ROS, thereby reprogramming the tumor immune microenvironment and promoting macrophage repolarization and cytotoxic T‐cell infiltration. In vivo studies further demonstrated robust inhibition of tumor growth and metastasis, together with durable antitumor immune memory following tumor resection, highlighting a complementary therapeutic paradigm for Hb‐based carriers.

While the first example targets RT, light‐activated therapies such as photothermal (PTT) and PDT require spatiotemporal control of oxygen availability and catalytic ROS generation. Gao et al. developed a plasmonic nanozyme system with integrated oxygen release and catalytic activity [13]. In this design, hollow porous Au nanocages (AuNCGs, ∼220 nm, pore size ∼40–50 nm) act as both catalytic nanozyme cores (plasmonic heating, catalytic ROS) and structural scaffolds for oxygen storage. Hb and perfluorohexane (PFO), a high oxygen solubility fluorocarbon, were co‐loaded into the AuNCGs, and pre‐saturated with oxygen. The hybrid core was then cloaked with murine breast cancer cell line (4T1) tumor cell membrane (CCM) pre‐anchored with the photosensitizer indocyanine green (ICG) to yield ICG@CCM‐AuNCG‐PO_2_‐Hb particles of ∼360 nm. This design offers homotypic targeting, immune cloaking, and combined PTT and PDT capability. Under 808 nm NIR irradiation, the AuNCGs exhibited a photothermal conversion efficiency of 26.8%, which is sufficient to vaporize the PFO and generate internal pressure (i.e., the transition temperature of the loaded PFO is around 58°C) that extruded the cell membrane through the pores, producing sub‐100 nm vesicles, hence the term “bubble nanomachine” [180]. The creation of smaller vesicles is an important fact that will allow for tumor penetration. Large NPs (>100 nm) accumulate well in tumors through the enhanced permeation and retention effect but penetrate poorly, whereas smaller ones (<100 nm) diffuse more deeply yet are rapidly cleared [181, 182, 183]. However, efficient nanomedicine delivery requires deep tissue penetration [184, 185], which is hindered by the abnormal TME characterized by irregular vasculature, high interstitial pressure, and a dense ECM (20–130 nm pores) that traps particles near blood vessels [186, 187, 188, 189, 190, 191, 192, 193, 194]. Hence, smart carriers capable of dynamic size reduction which are large for circulation and accumulation and then shrink in situ for deeper penetration, are highly desirable. Simultaneously, oxygen release from Hb and PFO increased dissolved oxygen from 5.8 mg L^−1^ (no NIR) to 8.4 mg L^−1^ under irradiation [195, 196]. Importantly, plasmonic heating not only triggered the PFO phase change but also endowed the AuNCGs with intrinsic peroxidase‐mimicking activity, accelerating ^1^O_2_ formation and thereby functioning as light‐activated catalytic nanozymes. Laser‐irradiated ICG@CCM‐AuNCG‐PO_2_‐Hb particles induced over 85% death in 4T1 tumor cells through homotypic targeting and synergistic photothermal‐photodynamic effects, while exhibiting much lower cytotoxicity toward normal NIH3T3 fibroblasts, confirming its tumor selectivity and biocompatibility. In vivo studies in 4T1 tumor‐bearing mice confirmed the strong therapeutic efficacy of the bubble nanomachines. Mice treated with ICG@CCM‐AuNCG‐PO_2_‐Hb particles plus NIR irradiation showed a 97% reduction in tumor volume, with complete tumor regression in 60% of cases, owing to enhanced tumor penetration of the laser‐triggered nanovesicles and the synergistic photothermal‐photodynamic effects. In contrast, treatment without irradiation showed negligible inhibition. Similar outcomes were observed in orthotopic tumor models, where most tumors were fully eradicated. Immunostaining further revealed markedly reduced HIF‐1α expression, indicating that oxygen release from the nanomachines effectively alleviated tumor hypoxia during therapy [13].

Beyond oxygen delivery and ROS amplification, recent work has further extended Hb‐based therapeutic carriers toward multigas transfusion strategies that exploit Hb's intrinsic catalytic activity. Li et al. engineered nano‐sized RBCs as dual gas carriers for carbon monoxide (CO) and NO, enabling amplified tumor gas therapy through Hb‐mediated redox catalysis [197]. Native RBCs were mechanically nano‐engineered by serial extrusion to yield ∼160 nm nRBCs retaining high Hb content and membrane integrity, and subsequently gas‐loaded to produce CO‐saturated (nRBC_CO_) or NO‐saturated (nRBC_NO_) constructs. Gas release was selectively triggered in tumor cells by elevated intracellular H_2_O_2_ levels, which oxidized Hb into metHb and disrupted gas coordination. In parallel, Hb functioned as a peroxidase‐like catalyst, converting H_2_O_2_ into O_2_^•^^−^ and ^•^OH, establishing a cascade amplification mechanism. In the combined nRBC_CO_/nRBC_NO_ treatment, mitochondrial stress and peroxynitrite (ONOO^−^) formation resulted in selective cancer‐cell apoptosis across multiple tumor models, accompanied by metabolic reprogramming and immune activation, while maintaining excellent biosafety.

Most recently, Hb itself has been re‐conceptualized as a controllable, biodegradable peroxidase‐like nanozyme for ferroptosis‐based cancer therapy, extending the oxygen–ROS amplification framework into a distinct redox modality [198]. Unlike RT‐ or PDT‐augmented HBOCs that rely on external activation, this strategy leverages tumor‐endogenous H_2_O_2_ to reactivate Hb's intrinsic catalytic activity. Wang et al. engineered a precision‐regulated nanoplatform in which CO poisoning temporally silences Hb's Fe–N_5_ active site during circulation, followed by tumor‐specific reactivation to trigger ferroptosis. Carboxyhemoglobin (HbCO) was immobilized within disulfide‐bridged dendritic mesoporous organosilica NPs (DMON) together with a Pt^4+^ cisplatin prodrug. In tumors, H_2_O_2_‐driven oxidation of heme iron induces CO desorption and restores catalytic activity, enabling ferric–ferryl redox cycling and lipid peroxide generation. Concurrent Pt^4+^ reduction to cisplatin increases intracellular H_2_O_2_ and depletes glutathione, downregulating GPX4 and establishing a feed‐forward ferroptotic amplification loop conceptually analogous to oxygen‐enhanced ROS cascades in RT and PDT systems. In vivo studies demonstrated pronounced tumor regression, extended survival, and minimal systemic toxicity.

Collectively, these examples illustrate the remarkable versatility of nanozyme‐integrated HBOCs in antitumor therapy. By coupling Hb‐mediated oxygen or gas transport with catalytic ROS/reactive nitrogen species generation, these systems can sensitize tumors to RT and PDT, enable deep tumor penetration and spatiotemporally controlled phototherapies, reprogram hypoxia‐driven immune suppression, or exploit tumor‐intrinsic redox chemistry to induce ferroptosis and gas‐mediated cytotoxicity. Across these diverse modalities, a unifying design principle emerges: precise control over when and where Hb's oxygen‐binding and catalytic functions are engaged allows hypoxia to be converted from a therapeutic obstacle into a targetable vulnerability, positioning Hb‐centric nanozyme platforms as a foundation for next‐generation precision cancer therapies.

## 6 Hb‐Related Nanozymes: From Antioxidant Shields to Catalytic Platforms for Diagnosis and Therapy

While Hb is classically exploited in artificial oxygen carriers for its oxygen‐binding capacity, its structural and catalytic versatility has inspired a new class of Hb‐related nanozymes. In these systems, Hb or RBC components no longer function as oxygen shuttles but instead serve as precursors, templates, or dopants that endow nanomaterials with enzyme‐mimicking or redox‐active properties. By integrating the intrinsic reactivity of the heme cofactor with nanostructured matrices, these constructs expand Hb's role beyond oxygen delivery toward ROS modulation, biosensing, and catalytic therapy [17]. Examples include how Hb‐derived motifs can be re‐engineered into multifunctional nanozymes for radioprotection, diagnostics, and infection control.

The development of nuclear technologies in medicine (e.g., computed tomography scans, RT) and other industries has increased the potential for human exposure to ionizing radiation [199, 200]. Ionizing radiation can produce copious amounts of ROS in biological tissues, and these ROS inflict damage on proteins, DNA, and cell membranes, leading to cell death or mutations. Protecting normal tissues from radiation‐induced injury is therefore a significant concern, especially for healthcare workers or patients undergoing RT. One approach to radioprotection is to scavenge the ROS generated by radiation before they can harm cells. The only FDA‐approved radioprotective drug, amifostine, acts via ROS scavenging but has serious side effects and limitations [201, 202]. Nanozymes, with their robust catalytic ROS‐neutralizing activity, have emerged as promising new radioprotective agents. Compared to small molecule antioxidants or enzymes, nanozymes offer better stability, bioavailability, and potential for targeted delivery. Wang et al. introduced an elegant Hb‐mediated green synthesis of ultrasmall MnO_2_ nanozymes for radioprotection [15]. In their one‐pot reaction, oxidizing potassium permanganate was reduced by Hb's amino and disulfide bonds under mild aqueous conditions, forming ∼5 nm NPs uniformly coated with fragmented Hb residues (Hb@MnO_2_NPs). The protein thus acted simultaneously as reducing, capping, and biocompatibilizing agent, eliminating the need for toxic reductants. MnO_2_ is a known nanozyme with potent peroxidase and CAT mimetic activity since it can decompose H_2_O_2_ and other ROS efficiently [203, 204, 205]. Indeed, the Hb@MnO_2_NPs showed broad spectrum ROS scavenging in assays, quickly neutralizing diverse radicals such as 2,2‐diphenyl‐1‐picrylhydrazyl (DPPH^•^), 2,2′‐azino‐bis (3‐ethylbenzothiazoline‐6‐sulfonic acid) (ABTS^•+^), ^•^OH, and O_2_^•−^, DPPH and ABTS radicals are among the most widely used model free radicals in laboratory assays for evaluating ROS‐scavenging activity, whereas ^•^OH and O_2_^•−^ are the two most biologically relevant free radicals in living organisms. This broad ROS scavenging ability is ideal for radioprotection, since radiation produces a burst of mixed ROS. The researchers tested the nanozyme in HUVECs, a model for radiation damage to blood vessels. Pretreating cells with Hb@MnO_2_NPs significantly reduced intracellular ROS levels after X‐ray irradiation, protected DNA from radiation‐induced double‐strand breaks and maintained much higher cell viability compared to untreated cells. Mechanistically, the nanozyme functioned as an in situ antioxidant catalyst, converting radiolytically produced H_2_O_2_ to benign products before oxidative cascades could propagate. Essentially, the nanozyme acted like an instant antioxidant shield inside cells during radiation exposure. Encouragingly, these benefits translated in vivo: mice that received Hb@MnO_2_NPs before being subjected to lethal whole‐body irradiation had a markedly prolonged survival time relative to control mice. The treated mice showed less oxidative damage in tissues and no observable toxic side effects from the nanozyme itself. This demonstrates that the Hb@MnO_2_NPs formulation can be considered an effective and safe radioprotective agent.

From a broader perspective, this study is notable for using Hb both as a material and a functional contributor: Hb's biochemical properties enabled the facile synthesis of a nanozyme, and remnants of Hb on the NP surface may also aid biocompatibility or functional synergy.

Nanozymes also hold great promise for biosensing, exploiting their catalytic oxidation of chromogenic substrates to transduce chemical information into optical or electrochemical signals [206, 207]. A classic example is using peroxidase‐mimicking nanozymes to catalyze the color change of chromogenic substrates (like TMB or ABTS) in the presence of H_2_O_2_. When integrated into a sensor device, such nanozymes can replace natural peroxidases (e.g., HRP), offering better stability, shelf life, and operational robustness, critical for point‐of‐care diagnostics.

Zhang et al. recently developed a Ru‐ and Hb‐co‐doped ZIF‐67 (Ru/Hb@ZIF‐67), for colorimetric cancer biomarker detection [16]. Their rationale stemmed from cancer metabolism: cancer cells uptake glucose at high rates and produce excess H_2_O_2_ both serving as metabolic hallmarks [208, 209, 210]. The Co‐based MOF ZIF‐67 inherently displays catalytic activity, but its efficiency is limited by electron‐transfer resistance and scarcity of active sites. Introducing Ru and Hb strategically modified the MOF's electronic microstructure and created abundant heteroatomic catalytic centers. During synthesis, Hb provided dispersed Fe atoms derived from its heme groups, while Ru served as a high‐valence redox co‐catalyst. This multi‐metal synergy (Co, Fe, Ru) promoted rapid electron shuttling between the substrate and oxidant. Structural analyses revealed that Fe incorporation prevented nanopore collapse and agglomeration, while Ru doping induced defect‐rich frameworks with enhanced conductivity. As a result, the Ru/Hb@ZIF‐67 composite displayed six‐fold higher peroxidase‐like activity than pristine ZIF‐67.

Using this nanozyme, the authors developed a straightforward colorimetric assay: in the presence of glucose, glucose oxidase (GOx) generates H_2_O_2_, which the nanozyme then uses to oxidize a chromogenic substrate, producing a visible color change. The color intensity correlates directly with the glucose or H_2_O_2_ concentration, allowing quantitative detection. This on‐site test could differentiate samples mimicking the metabolic profiles of cancer cells vs. normal cells by a quick visual readout. Such a tool could be used, for example, on tissue extracts or maybe even blood samples to flag abnormal metabolism associated with tumors.

Importantly, the incorporation of Hb into the MOF not only enriched catalytic diversity but also enhanced biocompatibility and hydrophilicity, facilitating integration into biological media. This strategy illustrates a broader design principle: biomolecular doping can tailor the catalytic and physicochemical landscape of MOF‐based nanozymes, merging inorganic precision with biological affinity. Beyond diagnostics, similar Hb‐modified MOFs could catalyze intracellular ROS modulation or oxygen evolution in hypoxic tumors, bridging sensing and therapy.

Whereas the previous example embeds Hb within a composite, Mohammadpour et al. showed that Hb itself can serve as the sole precursor for nanozyme formation [17]. By subjecting blood biowaste to a one‐step hydrothermal carbonization, they transformed the intrinsic Fe‐heme centers and organic matrix of Hb into Fe‐doped carbonaceous nanozymes without any added metal salts or ligands. This “waste‐to‐nanozyme” route exemplifies green chemistry and circular use of biomedical residues.

**FIGURE 7:**
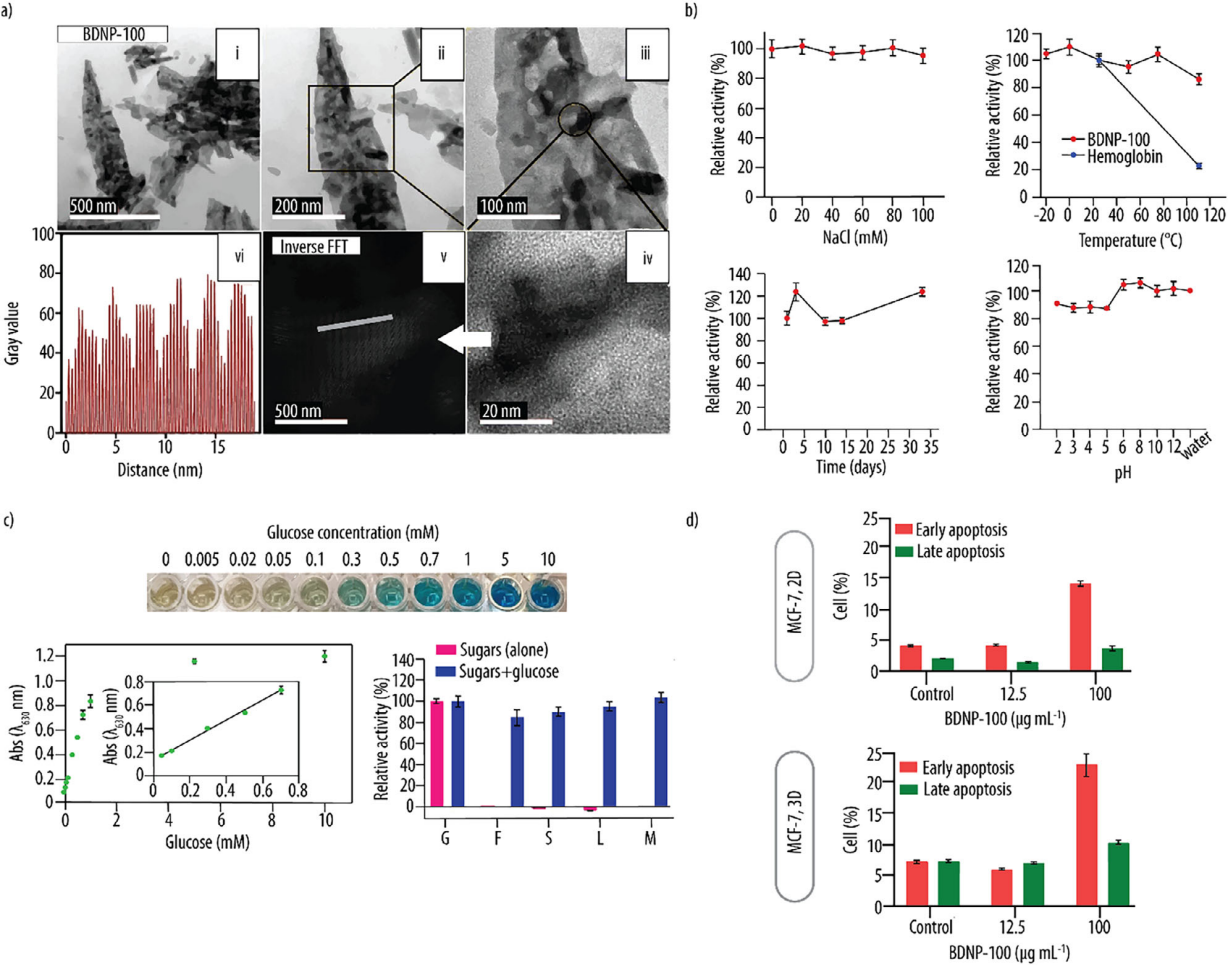
(a) Morphological analysis of blood‐derived nanoparticles (BDNP)‐100 with increasing magnification, (i–iii) employing high‐resolution TEM analysis (iv) before and (v) after applying inverse Fast Fourier Transform (FFT). (vi) The height profile of the grey line depicted in (v). (b) The effects of storage conditions (sodium chloride (NaCl), temperature, storage time, and pH) on the relative activity of BDNP‐100. (c) Photographic image and calibration plot of glucose against absorbance (Abs), and selectivity of the colorimetric assay towards different sugars individually (red bars) and co‐incubated with glucose (blue bars). G: glucose, F: fructose, S: sucrose, L: lactose, M: maltose. (d) Apoptosis rate of MCF‐7 cells in monolayer (2D, upper panel) and 3D spheroid model (3D, lower panel) treated with increasing concentrations of BDNP‐100. Adapted under CC‐BY 4.0 license [17].

The resulting blood‐derived NPs (BDNPs) displayed tunable physicochemical and catalytic properties depending on the reaction temperature (100°C–180°C). The authors suggested that under hydrothermal conditions, the combined effects of high temperature and pressure induced protein denaturation, followed by a cascade of chemical transformations including dehydration, pyrolysis, and double‐bond formation. These processes promoted the development of aromatic domains and subsequent nucleation, ultimately giving rise to fluorescent carbonaceous particles [211]. At 100°C, highly graphitic nanosheets (interlayer spacing ≈ 0.34 nm) were obtained, designated as BDNP‐100 (Figure 7a), which showed the strongest peroxidase‐like activity, whereas higher temperatures (≥150°C) yielded smaller, amorphous and fluorescent particles with diminished activity.

Kinetic assays using TMB and H_2_O_2_ established classic Michaelis‐Menten behavior, with yielded Michaelis‐Menten constants of 11.8 mm (for H_2_O_2_) and 0.12 mm (for TMB) and maximum reaction rates of 8.6 × 10^−8^ mol L^−1^ s^−1^ and 0.54 × 10^−8^ mol L^−1^ s^−1^, respectively. Despite containing roughly seven‐fold less Fe than conventional Fe‐doped carbon dots, BDNP‐100 showed superior catalytic efficiency, highlighting the critical role of the heme‐derived microenvironment. BDNP‐100 also retained 86% of their activity after heating to 110°C and remained stable across broad pH and ionic‐strength ranges (Figure 7b), highlighting the superior robustness of the nanozyme compared with Hb or natural peroxidases.

Functionally, BDNP‐100 integrated seamlessly into enzyme cascade systems. Coupled with GOx, it catalyzed the oxidation of TMB via the H_2_O_2_ generated from glucose, producing a quantifiable blue product. The linear detection range for glucose spanned 50–700 µm with a detection limit of 40 µm (Figure 7c) and a rapid 4‐min response, outperforming most reported colorimetric assays in sensitivity and speed. Beyond sensing, BDNP‐100's Fe‐based catalytic core could also amplify oxidative stress. In MCF‐7 breast‐cancer cells, BDNP‐100 combining with low exogenous H_2_O_2_ triggered pronounced apoptosis (Figure 7d), while sparing normal endothelial cells. This selective ROS‐mediated cytotoxicity suggests potential in chemodynamic or catalytic tumor therapy, where local H_2_O_2_ is harnessed to produce cytotoxic radicals in situ.

Collectively, the study demonstrated that the intrinsic architecture of Hb can be recast as a carbon nanozyme with dual antioxidant and pro‐oxidant functionality, depending on context. The facile, one‐pot fabrication requiring only waste blood and water paves the way for scalable, low‐cost production of Fe‐based nanozymes for biosensing, environmental catalysis, or redox medicine. Such up‐cycling of biological residues also aligns with sustainable manufacturing goals for nanotherapeutics.

**FIGURE 8:**
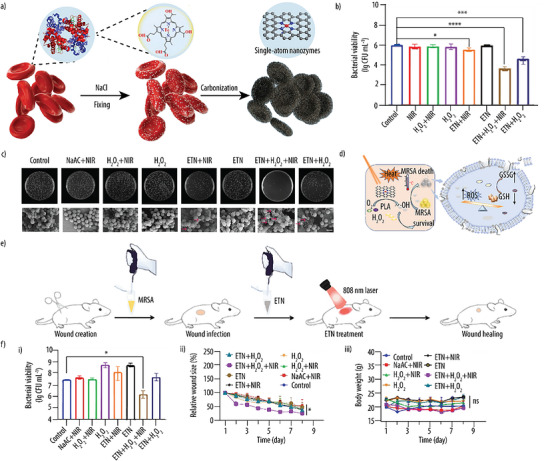
(a) Schematic illustration of erythrocyte‐templated nanozymes (ETN) with multifunctional properties for wound healing application. (b) The effect of various treatments on bacterial survival. NIR: near‐infrared, H_2_O_2_: hydrogen peroxide. (c) Photographic images of agar plates and respective SEM image of multi‐resistant *Staphylococcus aureus* (MRSA) treated with various treatments (scale bar = 1 µm). (d) Schematics of the putative antibacterial mechanism of ETN. PLA: peroxidase‐like activity, GSH: glutathione, GSSG: glutathione disulfide, ROS: reactive oxygen species. (e) Schematic illustration of establishing infected wound animal model and applying ETN treatment. (f) (i) Bacterial survival in mouse wound, (ii) relative wound size, and (iii) body weight of mice after applying various treatments. NaAC: sodium acetate. Adapted with permission [18]. Copyright 2023 John Wiley & Sons.

In chronic wounds, successful healing requires not only oxygen replenishment but also infection control and balanced redox signaling. While HBOCs can alleviate hypoxia, bacterial colonization and inflammation often hinder regeneration. Hb‐derived nanozymes can complement HBOCs by leveraging peroxidase‐like activity to generate bactericidal ROS locally. The erythrocyte‐templated nanozyme (ETN) approach epitomizes this concept. Here, RBC are leveraged as both structural templates and Fe sources to produce nanozymes with single‐atom dispersed Fe catalytic sites [18]. The rationale was that natural heme enzymes like HRP have a single Fe atom at their active center bound to four nitrogen atoms on a same plane (Fe‐N_4_) [212, 213]. If one could create a nanomaterial with similar single‐atom Fe‐N_4_ active sites, it might achieve very high peroxidase‐like activity (Figure 8a). RBCs are ideal templates because each erythrocyte is essentially a sack of ∼260 million Hb molecules, each containing a heme (Fe‐porphyrin) group [214, 215]. Since Hb is not preserved in its native form (its molecular architecture and oxygen‐binding capacity are lost during carbonization), Hb within the erythrocyte serves merely as a precursor for atomically dispersed Fe‐N_4_ catalytic sites embedded in a carbon matrix. Thus, the erythrocyte provides the chemical blueprint rather than the biological activity of blood. The result is a POD‐like catalyst capable of converting H_2_O_2_ into bactericidal radicals. Colorimetric assays (TMB, ABTS, or OPD confirm robust POD‐like activity, which is further enhanced by NIR via photothermal acceleration of reaction kinetics. Antibacterial efficacy is compelling. Against MRSA, ETN alone or in combination with either H_2_O_2_ or NIR produces modest effects; the triple combination (ETN + H_2_O_2_ + NIR) yields a 2.32‐log CFU reduction, indicating a strong synergistic interaction between peroxidase catalysis and photothermal heating (Figure 8b,c). Mechanistically, ETN treatment depletes bacterial glutathione and drives excess ROS production, pushing cells toward ferroptosis‐like death (Figure 8d). The effect is broad, occurring in both *S. aureus* and *E. coli*. In MRSA‐infected wounds (5 × 10^7^ CFU, 100 mm^2^ in Balb/c mice) (Figure 8e), ETN+ H_2_O_2_+NIR reduces bacterial burden and accelerates healing (Figure 8f (i)). By day 3, residual wound area is 26% vs. 38%–54% for controls, and by day 7 histology shows enhanced fibroblast proliferation and continuous epidermal coverage, while untreated wounds remain inflamed (Figure 8f (ii)). Additionally, no significant changes in mouse body weight were observed post‐treatment, indicating the absence of adverse reactions (Figure 8f (iii)). ETNs thus exemplify how Hb‐derived motifs can be re‐engineered as high‐performance catalytic nanozymes for infection control and pro‐healing microenvironment modulation, complementing oxygen‐focused HBOC strategies.

From a materials standpoint, ETNs demonstrate the power of biological templating to achieve atomic precision. The erythrocyte provides both a uniform geometry and a chemically pre‐organized heme distribution, facilitating the creation of single‐atom catalytic sites that rival natural enzymes in turnover efficiency. Moreover, the resulting nanozymes integrate seamlessly into photothermal or catalytic therapeutic regimens, enabling infection‐responsive, self‐sterilizing wound dressings that complement oxygen‐releasing HBOCs.

Taken together, these examples highlight how Hb and RBC‐derived architectures can be repurposed into versatile catalytic platforms spanning antioxidant defense, biosensing, and antimicrobial therapy.

## 7 Challenges and Limitations

Despite significant conceptual and technological advances, several challenges currently limit the clinical translation of nanozyme‐integrated HBOCs.

### 7.1 Oxygen‐Carrying Capacity

A central limitation is whether nanozyme‐HBOC formulations can achieve oxygen‐carrying capacities comparable to whole blood, where Hb concentrations reach ∼12–15 g dL^−1^. As summarized in Table 3, most reported systems operate at substantially lower effective Hb concentrations due to constraints imposed by colloidal stability, viscosity, and injectable volume. While this limitation is less critical for localized or adjuvant applications (e.g., wound healing or tumor therapy), it remains a major hurdle for large‐volume blood replacement and will require architectures enabling ultra‐high Hb loading or complementary transfusion strategies.

**TABLE 3:** Reported hemoglobin (Hb) concentrations of representative nanozyme‐integrated Hb‐based oxygen carriers designed as blood substitutes. Values are given in g dL^−1^ where available; in cases where direct Hb concentration was not reported, alternative metrics such as Hb mass or loading content (LC) are provided.

System	Reported [Hb]	Comment	Reference
Hb‐(HSA)_3_	5 g dL^−1^	—	10
AuNPs@PCN‐333‐Hb NCs	0.2 g dL^−1^	—	9
Hb@AuNCLs	1.3 g dL^−1^	—	11
PNPH	4 g dL^−1^	—	110
Hb@ZIF‐8 NPs	2.1 g dL^−1^	—	113
PLGA^Hb^‐NPs	—	LC: 22 ± 3%	96
PLGA/Hb^PDA^/(CeO_2_‐NPs)/PEG‐NCs	—	Hb mass: 150.3 ± 26.7 µg	8

Abbreviations: HSA: human serum albumin; AuNPs: gold nanoparticles; PCN: porous coordination network; NCs: nanocarriers; Hb@AuNCLs: Hb‐stabilized gold nanoclusters; PEG: polyethylene glycol; PNPH: polynitroxylated PEGylated Hb; ZIF‐8: zeolitic imidazolate framework‐8; PLGA: poly(lactic‐co‐glycolic acid); PLGA^Hb^: Hb‐loaded PLGA NPs; PDA: polydopamine; Hb^PDA^: PDA‐coated Hb; CeO_2_‐NPs: cerium oxide NPs.

### 7.2 Biosafety and Long‐Term Fate

Because many nanozyme‐integrated HBOCs rely on inorganic components (particularly metal‐based nanozymes) biosafety remains a critical and unresolved issue. Although short‐term biocompatibility is generally favorable, long‐term toxicity, biodegradation pathways, organ accumulation, and immunogenicity remain poorly understood, reflecting the broader infancy of the nanozyme field [216, 217, 218]. Comprehensive evaluation of cellular fate, pharmacokinetics (PK), biodistribution, metabolism, clearance, and delayed toxicity is essential, especially given that most nanozymes contain metal elements [218]. Such studies should extend beyond conventional small animal models and include systematic assessment of hemolysis, blood compatibility, and stability in complex biological fluids, which are particularly relevant for HBOC applications [218].

### 7.3 Catalytic Behavior Under Physiological Conditions

A further challenge lies in establishing clear structure–activity–function relationships for nanozyme‐HBOCs under physiologically relevant conditions. Nanozyme activity is highly dependent on atomic composition, crystal structure, particle size, and surface chemistry, yet most catalytic assessments are performed in simplified buffer systems [216, 217, 218]. In vivo, nanozymes encounter protein‐crowded environments, competing redox substrates, heterogeneous pH, and dynamic oxygen gradients, all of which may alter catalytic pathways. Distinguishing true catalytic cycling from sacrificial redox consumption, and identifying which specific enzyme‐like activities dominate biological outcomes (e.g., in multiactive systems such as CeO_2_), remain key mechanistic challenges [217].

### 7.4 Scalability, Recyclability, and Manufacturability

The structural complexity of many nanozyme‐HBOCs poses challenges for scalable, reproducible manufacturing under good manufacturing practice conditions. In addition, nanozyme aggregation, sensitivity to temperature or pH fluctuations, and limited recyclability during catalytic cycles may compromise both performance and cost‐effectiveness [218]. While recyclability is often discussed in the context of green chemistry, it is also relevant for reducing nanozyme dosage and cumulative exposure in vivo. Translation will therefore favor modular, robust designs with well‐defined composition, minimal batch‐to‐batch variability, and controlled degradation profiles.

## 8 Future Perspectives

Future research on nanozyme‐integrated HBOCs is expected to shift from proof‐of‐concept demonstrations toward precision‐engineered oxygen therapeutics, guided by deeper mechanistic understanding and translational considerations.

First, microenvironment‐responsive nanozyme‐HBOCs will play a central role. Systems capable of selectively activating oxygen release or catalytic activity in response to hypoxia, acidity, elevated ROS, or endogenous biochemical signals could dynamically adapt to disease‐specific conditions while minimizing systemic side effects [216, 218]. Expanding the repertoire of endogenous stimuli beyond simple redox triggers will improve response accuracy in complex physiological environments [216].

Second, rational design informed by structure–activity relationships will be essential. Elucidating how nanozyme composition, morphology, and surface chemistry govern catalytic behavior in vivo will enable selective enhancement of desired activities (e.g., SOD or CAT) while suppressing undesired ones (e.g., peroxidase or oxidase activity). Achieving both reaction specificity and substrate selectivity remains a major goal for advancing nanozyme‐HBOCs toward true enzymatic biomimicry.

Third, cross‐field integration with gene therapy and immunotherapy offers substantial opportunities. By alleviating hypoxia and modulating redox balance, nanozyme‐HBOCs can enhance gene delivery efficiency, normalize tumor vasculature, and reverse hypoxia‐driven immune suppression, positioning them as enabling platforms for combination therapies rather than stand‐alone agents.

Fourth, personalized dosing and pharmacodynamic (PD) optimization represent an emerging frontier. Establishing therapeutic windows, minimal effective doses (MED), maximum tolerated doses (MTD), and time‐dependent efficacy profiles will be critical for correlating nanozyme catalytic activity with therapeutic outcomes. Such studies must move beyond single‐dose or single‐time‐point evaluations to include systematic PK/PD analyses and multi‐model validation.

Fifth, materials evolution toward biodegradable and biomimetic nanozymes will be key to long‐term safety and regulatory acceptance. Hb‐derived carbon nanozymes, single‐atom catalysts, and degradable MOF‐based platforms offer promising routes to retain catalytic functionality while reducing long‐term accumulation and immunogenicity risks [218]. Surface functionalization strategies (i.e., biomimetic coatings, PEGylation, and zwitterionic materials) will further enhance circulation time and biocompatibility.

Finally, translation will require rigorous in vivo validation and regulatory alignment. Although nanozyme‐integrated HBOCs have shown compelling efficacy in preclinical wound‐healing and cancer models, their evaluation has so far been limited to animal studies, leaving a substantial gap to human clinical application. Addressing ethical, environmental, and regulatory considerations—including sustainable disposal and standardized evaluation frameworks—will be essential for responsible development and eventual clinical translation.

## 9 Conclusion

Nanozyme‐integrated HBOCs represent a new generation of oxygen therapeutics that move beyond passive oxygen delivery toward functional biomimicry of RBCs. By coupling Hb's reversible oxygen binding with catalytic redox regulation, these systems directly address one of the most persistent limitations of conventional HBOCs, namely, the oxidative instability of cell‐free Hb‐ and transform oxygen carriers into self‐protecting, multifunctional platforms.

Across diverse material classes, including metallic nanozymes, MOFs, carbon‐based catalysts, and Hb‐derived systems, nanozyme incorporation has been shown to preserve Hb in its ferrous state, suppress metHb formation, and mitigate ROS‐induced toxicity through catalytic rather than consumptive mechanisms. These advances are particularly relevant given that, to date, the majority of reported nanozymes catalyze redox reactions associated with ROS generation (e.g., peroxidase‐ and oxidase‐like activities), whereas comparatively few exhibit ROS‐eliminating functions such as SOD‐ or CAT‐like activity, which are critical for HBOC stabilization [216]. In this context, nanozyme‐HBOCs constitute a strategically important subset of nanozyme systems that prioritize redox homeostasis rather than oxidative amplification.

Beyond blood substitution, these properties enable broad biomedical utility. In wound healing, nanozyme‐HBOCs restore local oxygenation while simultaneously modulating inflammation, redox balance, and infection. In oncology, they convert tumor hypoxia from a therapeutic barrier into a targetable vulnerability, enhancing RT, PTT, PDT, and emerging redox‐driven modalities. Importantly, recent advances extending HBOCs toward CA‐like activity highlight a shift from single‐function oxygen carriers toward multigas, regulatory oxygen therapeutics, suggesting that future artificial blood systems may partially reproduce the integrated gas‐exchange and regulatory functions of native erythrocytes.

## Conflicts of Interest

The authors declare no conflicts of interest.

## Data Availability

The authors have nothing to report.
